# Systematics and biology of the new genus *Macrosaccus* with descriptions of two new species (Lepidoptera, Gracillariidae)

**DOI:** 10.3897/zookeys.98.925

**Published:** 2011-05-13

**Authors:** Donald R. Davis, Jurate De Prins

**Affiliations:** 1 Department of Entomology, National Museum of Natural History, Smithsonian Institution, P.O. Box 37012 MRC 105, Washington, D.C. 20013-7012, U.S.A.; 2 Department of African Zoology, Royal Museum for Central Africa, Tervuren, Belgium

**Keywords:** Biogeography, DNA barcodes, host plants, hypermetamorphosis, genital morphology, larval morphology, Lithocolletinae, pupal morphology, leaf mining, taxonomy

## Abstract

The new genus *Macrosaccus* Davis & De Prins is proposed for three species formerly assigned to the genus *Phyllonorycter*: *Macrosaccus robiniella* (Clemens), *Macrosaccus morrisella* (Fitch), and *Macrosaccus uhlerella* (Fitch); two new, closely related species: *Macrosaccus neomexicanus* Davis and *Macrosaccus gliricidius* Davis, are also proposed. Descriptions of the adults, pupae, larvae, life histories, and distributions are supplemented with photographs, line drawings, and scanning electron micrographs. Larvae of all species are serpentine/blotch leaf miners on various genera of the plant family Fabaceae. The genus is endemic to the New World, with the invasive species *Macrosaccus robiniella* now widely established in Europe.

## Introduction

The gracillariid subfamily Lithocolletinae includes 503 species (De Prins and De Prins 2011) assigned to 7–9 genera according to different authors (Kumata 1993; Davis and Robinson 1998; Kuznetzov and Baryshnikova 2001). Lithocolletinae are very small moths (less than 10 mm in wing expanse) with often brilliantly coloured forewings of ochreous-orange or reddish-brown ground colour, marked by white or silvery white striae and fasciae (Chapman 1902; Vári 1961; Kumata 1961, 1963, 1993; Watkinson 1985; Kuznetzov 1981; Kuznetzov and Stekol’nikov 1987, 2001; Davis and Robinson 1998; Kuznetzov and Baryshnikova 2001). The subfamily has a worldwide distribution, but is more species-rich in temperate zones: 273 species are known from the Palaearctic, 142 species from the Nearctic, 49 species from the Oriental, 26 species from the Afrotropical, 16 species from the Neotropical, and only 8 species from the Australasian region (De Prins and De Prins 2005, 2011). The hostplant range within this subfamily is broad. Lithocolletinae feed on no less than 36 families of plants (De Prins and De Prins 2005, 2011). Approximately 32 families of dicotyledonous plants serve as hosts for *Phyllonorycter* moths, compared to 11 families for *Cameraria* (De Prins and De Prins 2005, 2011). Deschka (1993) lists 13 hostplant families for *Cameraria*, but we failed to find any published record related to *Cameraria* larvae feeding on either Oleaceae or Sapotaceae. Seven lithocolletine genera (*Cameraria*, *Phyllonorycter*, *Chrysaster*, *Hyloconis*, *Neolithocolletis*, *Porphyrosela* and *Protolithocolletis*) mine plants of the family Fabaceae, with the latter five genera feeding exclusively on Fabaceae (Robinson et al. 2007; De Prins and De Prins 2011). Probably many more taxa will be discovered in the southern hemisphere. However, even in the seemingly well-known European and North American Lithocolletinae fauna the generic assignment of some lithocolletine species is still questionable; for example, the species-rich genus *Phyllonorycter*, comprising about 400 species and having a world-wide distribution, has served for some time as a depository for several species of uncertain phylogenetic placement.

Characters defining genera within the Lithocolletinae are still being investigated. Most of these concern the life history and morphology of the preimaginal stages (Kumata 1993). With regards to adult morphology, Kumata (1993) diagnosed Lithocolletinae genera by the following characters: 1) wing venation, in particular the parallel condition of veins Rs and M near the base of the hindwing; 2) number of setae on apical part of tegumen in male genitalia; 3) development of the transtilla in male genitalia; 4) number and shape of signa in female genitalia; and 5) dark margins of whitish fasciae on forewings. Adult morphology does not always clearly separate genera: for example, 1) the wing venation in *Phyllonorycter* and *Cameraria* is identical (with the exception of *Cameraria fasciata*); 2) a pair of setae on the apical part of the tegumen is present in *Cameraria*, *Chrysaster* and *Porphyrosela* (absent in *Phyllonorycter*); more than two pairs of tegumenal setae are found in *Hyloconis* and *Cremastobombycia*; 3) transtilla is incomplete in *Hyloconis* and *Cameraria* (with the exception of *Cameraria magnisignata*, *Cameraria milletiae*, *Cameraria palawanensis*, C*. pongamiae*, and *Cameraria virgulata*); 4) signa of the corpus bursae show a variety of shapes in *Phyllonorycter*; 5) most of the species in both *Cameraria* and *Cremastobombycia* show black margins distally on the whitish fasciae (Busck 1909; Ely 1918; Braun 1925; Kumata 1963, 1993, 1995; Opler and Davis 1981; Powell and Opler 2009). Recently, a multidisciplinary approach was undertaken which incorporates adult morphology, chemical communication and DNA barcoding to resolve the generic assignment within Lithocolletinae (De Prins et al. 2009). Although this approach appears useful to assess the generic limits of Gracillariidae, it is applicable only to those few groups of species for which chemical communication has been studied (De Prins et al. 2009; Liblikas et al. 2009 and the references therein). Collecting efforts are beginning to reveal the existence of several previously unknown Lithocolletinae genera in the tropics. An evaluation of generic apomorphies will only be accomplished after a thorough phylogenetic study of Lithocolletinae genera, based on both molecular and morphological characters, is completed.

The purpose of this paper is to propose and diagnose a new lithocolletine genus, *Macrosaccus*, and to document the five species we recognize within this New World group. This study is long overdue because one species, *Macrosaccus robiniella* (Clemens), has become a serious invasive pest on the introduced *Robinia pseudoacacia* L. (Fabaceae) over much of Europe. With this contribution we also attempt to broaden the understanding of the generic definitions within Lithocolletinae. We transfer three previously known *Phyllonorycter* species to *Macrosaccus*, clarify the synonymy, and designate lectotypes whenever possible for the species-group taxa. Additionally, we provide DNA barcodes as identification aids and descriptions of two new congeneric species which also were reared from Fabaceae.

## Methods

*Collecting and rearing.* Field investigations were carried out in Europe (Belgium), Canary Islands (La Palma), and in several states within the United States (Arizona, Illinois, Maryland, New Mexico). All specimens examined in this study were reared from species of Fabaceae which are summarized in Table 1.

Leaves containing mines with larvae were placed in plastic bags or rearing containers periodically moistening the lids protecting the specimens from drying out. Specimens were pinned, spread and mounted in the usual way for morphological examination. Some voucher samples of reared specimens were fixed in 100% ethanol for DNA analysis. Larvae and pupae collected on *Robinia pseudoacacia* L. were preserved in 75% ethanol.

**Table 1.** *Macrosaccus* and four *Phyllonorycter* species that feed on Fabaceae.

*Moth species*	*Host plant species*	*Country*	*Reference*
*Macrosaccus gliricidius* Davis, sp. n.	*Gliricidia sepium* (Jacq.)	Guadeloupe, Honduras	Present study
*Macrosaccus morrisella* (Fitch, 1859)	*Amphicarpa bracteata* (L) Fernald	Canada	Present study
*Macrosaccus morrisella* (Fitch, 1859)	*Amphicarpa bracteata* (L) Fernald	U.S.A.	Chambers 1878: 111
*Macrosaccus morrisella* (Fitch, 1859)	*Amphicarpa* sp.	Canada	Present study
*Macrosaccus morrisella* (Fitch, 1859)	*Strophostyles leiosperma* (Torrey & A. Gray)	Canada	Present study
*Macrosaccus neomexicanus* Davis, sp. n.	*Robinia neomexicana* Gray	U.S.A.	Present study
*Macrosaccus robiniella* (Clemens, 1859)	*Robinia hispida* L.	U.S.A.	Chambers 1878: 111
*Macrosaccus robiniella* (Clemens, 1859)	*Robinia pseudacacia* L.	Belgium	De Prins and Groenen 2001: 159
*Macrosaccus robiniella* (Clemens, 1859)	*Robinia pseudacacia* L.	U.S.A.	Clemens 1859: 320.
*Macrosaccus robiniella* (Clemens, 1859)	*Robinia viscosa* Vent	U.S.A.	Chambers 1878: 111
*Macrosaccus uhlerella* (Fitch, 1859)	*Amorpha fruticosa* L.	U.S.A	Chambers 1878: 110
*Phyllonorycter cytisifoliae * (M. Hering, 1927)	*Chamaecytisus proliferus* (L.) Link	Canary Islands: La Palma	Hering 1927: 419
*Phyllonorycter foliolosi* Walsingham, 1908	*Adenocarpus foliolosus* (Ait.) DC	Canary Islands: La Palma	Walsingham 1908: 978
*Phyllonorycter medicaginella* (Gerasimov, 1930)	*Medicago sativa* L.	Belgium	Kuchlein et al. 2002: 89
*Phyllonorycter nigrescentella* (Logan, 1851)	*Vicia sepium* L.	Belgium	Fologne 1862: 24

### Morphology

Adults were examined externally using either MZ12.5 or Nikon SMZ 1500 stereomicroscopes (maximum magnification 200×). Genitalia were prepared following Robinson (1976) with some modifications. After maceration of the abdomen in 10% KOH for 24 hours or by heating in hot 10% KOH for ~ 30 minutes, and subsequent cleaning and deionization, the male genitalia were stained with 2% eosine B, a mixture of 2% azophloxine and 2% acid fuchsin; the female genitalia were stained with a 1% chlorazol black E solution and embedded in Euparal or Canada balsam. Genital morphology was examined using a Leica DMLB microscope under magnifications of 150×, 200×, and 400×. The terminology follows Vári (1961), Klots (1970), (Kumata (1993, 1995), and Kristensen (2003). Microslides for studies of wing venation were prepared following the technique suggested by Vári (1961) and applying modifications used by Hoare (2000). Some wing slides were cleaned, stained with saffranin, and mounted dry beneath a glass coverslip.

For scanning electron microscopy, the immatures were immobilized by moment freezing at -27°C. Pupae were sputtered-coated with gold using a Bal-TEC/SCD 005 Sputter Coater. The images were taken with a Jeol MP 35060 camera combined with a Jeol JSM-5400 LV Electron Scanning Microscope and processed using the Orion 4 High Resolution Image Grabbing System software. Larval terminology follows Davis (1987).

The spellings of all species names were retained as originally proposed.

### Molecular analysis

**Figure 1.**
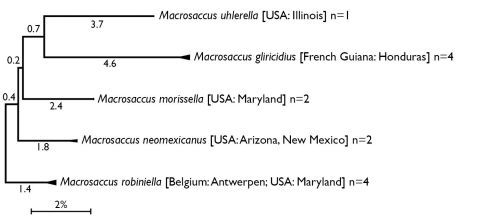
Compressed subtree sequenced data for cytochrome c oxidase I (COI) of *Macrosaccus*, derived from 13 samples among 5 species based upon neighbor-joining analysis with Kimura 2-parameter model. Numbers above branches indicate branch length. Sequence lengths obtained for all samples were 658bp each.

Sequences of the 658bp Cytochrome Oxidase I were generated at the Biodiversity Institute of Ontario, University of Guelph, Canada. DNA was extracted from legs or entire bodies of adult moths using a QIAGEN DNeasy Extraction Kit (Qiagen, Inc., Valencia, CA). Primers LepF1 and LepR1 (Herbert et al. 2004) were used to obtain the barcoding fragment of COI following methods previously described (Hajibabaei et al. 2006). Sequences are available at the National Center for Biotechnology Information GenBank database and at the Barcode of Life Database (BOLD). A neighbor-joining (NJ) tree was generated utilizing the Kimura 2-parameter model via the BOLD website (http://www.boldsystems.org/; Ratnasingham and Hebert 2007) to illustrate the genetic divergences between species. A compressed version of this tree was produced (Fig. 1) using Molecular Evolutionary Genetics Analysis (MEGA) version 4 (Tamura et al. 2007).

**Table 2.** Mitochondrial DNA (COI) sequence divergence (%) among species of *Macrosaccus*. Uncorrected average pairwise distances are shown for the barcoding region of (COI). Shaded cells contain means within species distances. Cells below shaded diagonal contain mean between species distances. Species abbreviations in the heading refer to species listed in left column.

	gliri	morr	robi	neom	uhle
*Macrosaccus gliricidius*	0.4				
*Macrosaccus morrisella*	8.1	0.0			
*Macrosaccus robiniella*	8.1	4.7	0.4		
*Macrosaccus neomexicanus*	7.1	4.5	4.1	0.5	
*Macrosaccus uhlerella*	8.6	6.7	6.5	7.3	0.0

### Museum collections

Whenever possible the primary types of every species were examined. Lectotypes were designated from the syntypic series whenever available.

Abbreviations of Institutions from which specimens were examined are:

ANSP Academy of Natural Sciences, Philadelphia, Pennsylvania, USA.

BMNH The Natural History Museum (formerly the British Museum (Natural History)), London, United Kingdom.

CCDB Canadian Centre for DNA Barcoding, University of Guelph, Canada

CNC Canadian National Collections of Insects, Arachnids and Nematodes, Agriculture and Agri-Food Canada, Ottawa, Canada.

CU Cornell University, Ithaca, New York, USA.

INHS Illinois Natural History Survey, Champaign, Illinois, USA.

MCZ Museum of Comparative Zoology, Harvard University, Cambridge, Massachusetts, USA.

RMCA Royal Museum for Central Africa, Tervuren, Belgium

**USNM** Collections of the former United States National Museum, now deposited in the National Museum of Natural History, Smithsonian Institution, Washington, D.C., USA.

## Systematic Account

### Macrosaccus

Davis and De Prins gen. n.

urn:lsid:zoobank.org:act:2451DAED-FEB2-4E03-B86C-88F10584A067

http://species-id.net/wiki/Macrosaccus

#### Type species:

*Lithocolletis robiniella* Clemens, 1859, by original designation.

*Macrosaccus* is assigned to the subfamily Lithocolletinae on the basis of the following putative morphological synapomorphies: hindwing vein Rs parallel to vein M and costal margin; adults rest with body parallel to surface; adult head with occipital tuft; and pupation occurring within the mine.

#### Diagnosis.

Superficially, *Macrosaccus* is similar to nearly all other genera of Lithocolletinae, sharing such characters as a well developed occipital tuft; a forewing pattern accentuated with oblique, whitish strigulae; and by the mode of pupation which occurs inside a silken cocoon within the whitish blotch mine usually on the underside of the host leaf without any prepared exit opening. However, in contrast to the typically solitary larvae and pupae of other Lithocolletinae genera, those of *Macrosaccus* are often gregarious inside a single, composite mine. The wing venation of *Macrosaccus* is similar to that of *Cameraria* and *Phyllonorycter* in possessing five apical veins, but it differs from the two latter genera in having Rs4 rising either from the base of Rs3 or stalked with Rs3. The hindwing venation is similar to *Cameraria*, *Chrysaster*, *Leucanthiza*, *Neolithocolletis*, and *Phyllonorycter*, but differs from *Cremastobombycia*, *Hyloconis*, *Porphyrosela*, and *Protolithocolletis* in the absence of vein M2. In the male genitalia, the sternum 8 is not produced caudally as in *Chrysaster*, *Leucanthiza*, and *Protocolithocolletis*. In *Cameraria*, *Cremastobombycia*, *Hyloconis*, *Neolithocolletis*, *Phyllonorycter*, and *Porphyrosela*, the sternum 8 forms a large flap underlying the valvae. The apex of the tegumen in *Macrosaccus* possesses a pair of tiny setae as in *Cameraria*, *Chrysaster* and *Porphyrosela*, but unlike *Phyllonorycter* which lacks apical setae. The transtilla of *Macrosaccus* is complete like that of other lithocolletine genera, but it differs from that of *Cameraria* and *Hyloconis* where it is incomplete. The female genitalia of *Macrosaccus* are characterized by numerous, microscopic spine-like signa which are scattered within the subcaudal part of corpus bursae (in other lithocolletine genera the corpus bursae bears other types of signa). Though the adult head of *Macrosaccus* is very similar to that of *Protolithocolletis*, the venation between these two genera differs with the forewing of *Protolithocolletis* more developed in possessing veins Rs1 and M2. The pupae provide perhaps the best characters for generic distinction, with that of *Protolithocolletis* lacking the spinose accessory cremaster ridge on sternum 7, which is characteristic for *Macrosaccus*.

#### Adult.

*Head* (Figs 10, 11). Vertex covered with long dense tuft of piliform scales; frons with smooth appressed scales; eyes of midsize; interocular index (= vertical eye diameter/interocular distance) ~ 0.75–0.96. Antenna about 0.7× the length of forewing (n=9), smooth scaled, with a single row of scales per segment; scape with dense pecten. Proboscis well developed, naked, ca. 1.8–2.5× length of labial palpus. Maxillary palpus very short, rudimentary, ~ 0.5× length of labial palpomere II, and directed laterally; consisting of 2 articulated segments; basal 2 segments fused; segment 3 free, spherical. Labial palpus slender, drooping, with ratio of segments from base 1.5: 1: 2.

*Thorax* (Fig. 12). Forewing slender, maximum width/length ratio ca. 0.2, narrow at apex. Venation with 8 veins, apical part with 5 veins; Sc strong, extending nearly to costa, basal half of R indistinct, Rs2 present, Rs3 arises from apex of the cell, position of Rs4 variable, arising either from base of Rs3 or stalked with Rs3, M and CuA1 separate, CuP indistinct (fold) for entire length, 1A strong, separate, discal cell either open (with absence of crossvein between Rs2 and Rs3) or closed, extending ~ 0.78 of wing length. Hindwing lanceolate, maximum width 0.12 that of length, venation reduced, similar to *Phyllonorycter*; Sc very short, Rs very long, extending almost to apex; basal 2/3 of M indistinct, parallel to Rs, distal part of M ends near distal 3/5: basal half of Cu strong, distal half indistinct, ending slightly before midway along dorsum; frenulum a single stout bristle in male, 2 tightly appressed bristles in female, retinaculum in male consisting of a broadly triangular curved fold from the ventral base of Sc and a few stiff, forward directed scales situated on the posterior part of Cu.

*Abdomen*. The margins of the abdominal opening strongly sclerotized and broad laterally, the sclerotized margination of abdomen opening unconnected on T2, S2 apodemes long, ~ half the length of S2, generally slender but more stout at basal 1/3 and very slender at distal 2/3; two pairs of tiny spinules on S2 sublaterally, and a pair of tiny spinules on S3–S6 sublaterally. Sternum 8 in male undeveloped.

*Male genitalia*. Tegumen relatively short, broad, moderately sclerotized laterally. Caudal portion covered with numerous tiny setae. A pair of long, slender setae present at apex of tegumen. Vinculum broad, U-shaped with very slender, elongate saccus which ranges from 1.1–1.7× the length of valva. Valvae symmetrical, moderately broad, costal margin nearly straight to slightly curved; ventral margin variable between species from slightly convex to slightly concave over distal half with apex varying from fully round to abruptly narrowing; median surface of valva with sparse setae of medium length; apex of valva densely covered with longer, more stout setae. Transtilla complete and well developed, laterally expanded into rounded lobes. Aedeagus very long, nearly as long as entire genital capsule (from apex of tegumen to anterior end of saccus), straight and slender, of uniform diameter along its length; caudal end of vesica usually with long, slender cornuti; phallobase ~ ¼ total length of aedeagus.

*Female genitalia*. Papillae anales flattened, strongly interconnected, covered with short setae mostly along apical margin; basal bar broad but weakly sclerotized. Posterior apophyses slightly longer than width of papillae anales, straight and slender. Segment 8 short, weakly sclerotized. Anterior apophyses as long or slightly shorter than posterior apophyses, with moderately broad bases, then slender extending to caudal 1/3 of segment 7. Ostium bursae opens medially, near caudal margin of segment 7; sterigma simple, without cuticle folds, antrum funnel-shaped, narrowing anteriorly. Subcaudal area of segment 7 mottled with numerous tubercles. Ductus bursae ~ 2× times longer than segment 7; a membranous accessory bursae ~ 2/3 the length of corpus bursae, arising from middle to anterior 1/3 of ductus bursae, with a smaller lateral pouch arising ~ midway along side of accessory bursae. Corpus bursae 1.0–2.0× the length of segment 7, subcaudal region of corpus bursae usually with scattered spicules or with spicules arranged in linear rows in *Macrosaccus robiniella*.

#### Larva.

Hypermetamorphic with five larval instars. Earliest instars (1–3) highly modified sapfeeders with strongly depressed bodies and reduced chaetotaxy; 3 pairs of stemmata arranged in a lateral, anterior cluster on head; labrum short and broad, bilobed; anterior margin broadly concave, roughened, with minute dentations along inner margin of lateral lobes; maxillary and labial palpi absent. Later instars (4 and 5) tissue feeders, with cylindrical bodies. Head approximately round with full complement of mouthparts; 4 pairs of stemmata present; antenna 3-segmented with first segment moderately long; labrum strongly bilobed with raised median portion on each lobe; M1 absent; numerous secondary spines visible from inner, ventral perimeter of labrum. Thorax with SD1 elongate, immediately ventral to XD2; SD2 absent on T1, present on T2–3L group bisenose on T1–3. SV unisetose on T1–3. Legs relatively short but fully developed; coxae widely separated, with 4 coxal setae. Abdomen with D and SD groups bisetose on A1–8, 10; unisetose on A9; L group bisetose on A1–5, unisetose on A6–10; prolegs present on A3–5, 10; crochets of A3–5 arranged in a uniordinal circle; anal proleg with crochets arranged in a uniordinal semicircle opened caudally; anal plate with 4 pairs of setae.

#### Pupa.

Head with vertex terminating in a relatively short, broadly triangular, acute frontal process (cocoon cutter). Abdomen mostly covered dorsally and ventrally with dense, minute spines; dorsum of A2–7 with a single anterior row of short, stout spines; caudal half of sternum 7 with a transverse ridge (accessory cremaster) bearing ~ 18–21 mostly longitudinal rows of short, blunt spines; cremaster of A10 greatly reduced, nearly absent, consisting of 1–2 pairs of minute tergal spines.

#### Etymology.

The generic name is derived from the ancient Greek μακρο- (long) and σάκκος (bag) in reference to the elongate saccus in the male genitalia. Gender masculine.

#### Generic relationships and species diversity.

Several morphological specializations closely associate *Macrosaccus* with the genera *Chrysaster*, *Cremastobombycia*, and *Phyllonorycter*. Some of these involve the moderately produced proboscis (~ 2× the length of the labial palpi) and the very reduced, two-segmented (with basal segment relatively enlarged), broad maxillary palpi (Figs 10, 11). The wing venation of all three genera is nearly identical and is among the most reduced within Gracillariidae. Only three branches of Rs are present in the forewing, accompanied by single branches of M and Cu (Fig. 12). Venation in the lanceolate hindwings is similarly reduced with only three major veins usually preserved (Rs, M, and Cu) in addition to the extremely basal Sc+R1. The position of Rs4 in *Macrosaccus* differs somewhat from that in the aforementioned three genera in arising either from the base of Rs3 or stalked with Rs3. Perhaps more significantly is that the discal cell is usually open in *Macrosaccus* due to the total or near absence of the Rs2-Rs3 crossvein. This crossvein is usually present in the other genera.

The most distinguishing feature in the male genitalia of *Macrosaccus* is the extremely long, rodlike saccus, whence the generic name is derived. The male saccus in *Chrysaster*, *Cremastobombycia*, and *Phyllonorycter* is either undeveloped or much shorter and stouter (except in two Afrotropical species *Phyllonorycter farensis* and *Phyllonorycter obandai*). Likewise sternum 8 in all males of these three genera is extended caudally as a variably lengthened plate beneath the genitalia, compared to being unmodified in *Macrosaccus*. The female genitalia of *Macrosaccus* typically possess a relatively large, variably shaped accessory bursa arising approximately midway along the long, slender ductus bursae. The corpus bursae contains dense patches or faint rows of minute spines. The accessory bursae in *Phyllonorycter* originates more caudally near the ostium, and usually two, circular and variably sclerotized signa are present (Davis and Deschka 2001).

The pupa of *Macrosaccus* is characterized by an accessory cremaster on abdominal sternum 7 that is unlike that of any other known gracillarid genus. This consists of a raised transverse ridge bearing ~ 18–21 mostly longitudinally oblique rows of short, blunt spines (Figs 84, 85). The accessory cremaster when present in *Phyllonorycter* differs greatly in consisting of a raised triangular area located midventrally on sternum 7 with 1–2 pairs of stout spines projecting laterally (Davis and Deschka 2001).

In addition to the foregoing morphological characters, a preliminary molecular phylogeny based on ten genes also strongly places *Macrosaccus* apart from *Phyllonorycter* (Kawahara 2010). Morphological characters distinguishing *Macrosaccus* from *Phyllonorycter* are summarized in Table 3.

Five species, all indigenous to the New World, are currently recognized in the new genus *Macrosaccus*. The high sequence divergence of the barcoding region of COI (> 7%) between species (Fig. 1, Table 2) further confirms the species concepts previously determined by morphological and larval host information. Sequence divergences within species for the 12 samples with multiple specimens were low and varied between 0–0.62% (*Macrosaccus gliricidius*), 0% (*Macrosaccus morrisella*), 0–0.46% (*Macrosaccus neomexicanus*), and 0–0.71% (*Macrosaccus robiniella*). The latter included specimens from Belgium and the United States.

**Table 3.** Diagnostic comparisons between adults and pupae of *Macrosaccus* and *Phyllonorycter*.

Character	Macrosaccus	Phyllonorycter
Sternum 8	Unmodified (not extended)	Caudally extended
Male genitalia: apex of tegumen	With 2 setae	No setae
Male genitalia: saccus	Saccus longer than valva in all species	Saccus shorter than valva except in two Afrotropical species
Male genitalia: setation of valva	Only apex of valva densely covered with elongate, stout setae	Other types of setation
Male genitalia: aedeagus	ca. 2× as long as genital capsule from apex of tegumen to anterior end of vinculum	Significantly shorter except in three Afrotropical species
Female genitalia: signum	Consisting of numerous microscopic spicules scattered or in linear series on subcaudal part of corpus bursae	Signa not scattered, often confined to 1–2 moderately sclerotized, oval areas
Forewing venation	Rs4 arises either from base of Rs3 or is stalked with Rs3	Rs3 and Rs4 separate
Pupa: accessory cremaster of sternum 7	An elongate, transverse ridge bearing 18-21 oblique rows of minute spines	No transverse ridge; instead located midventrally, with 1-2 pairs of lateral spines

## Key to species of Macrosaccus (based primarily on male genitalia and larval host)

**Table:** 

1	Valva of male of uniform width to broadly rounded apex (Fig. 32); host *Gliricidia sepium*	*Macrosaccus gliricidius*
–	Male valva narrowing before apex	2
2	Valva gradually tapering to narrow apex (Fig. 23); host *Robinia neomexicana*	*Macrosaccus neomexicanus*
–	Valva constricted before apex	3
3	Valva strongly constricted at middle (Fig. 28); distal half less than half the width of sacculus; host *Amorpha fruticosa*	*Macrosaccus uhlerella*
–	Valva slightly constricted near apex	4
4	Forewing with a short, oblique white streak from base of costa; median white fascia complete, slightly curved outward (Fig. 5). Valvaconstricted before apex; arms of transtilla reduced (Fig. 18); hosts *Amphicarpa bracteata*, *Strophostyles leiosperma*	*Macrosaccus morrisella*
–	Forewing without basal white costal streak; median white fascia usually broken, strongly oblique (Figs 2–4). Valva constricted closer to apex; arms of transtilla broader (Fig. 13); hosts *Robinia pseudoacacia*, *Robinia hispida*, *Robinia viscosa*	*Macrosaccus robiniella*

### Macrosaccus robiniella

(Clemens) comb. n.

http://species-id.net/wiki/Macrosaccus_robiniella

Figs 1 4 10 17 36 40 59 98 Tables 1 2 4 5

Lithocolletis robiniella Clemens 1859 (Nov.): 318; 1872: 66.- Chambers 1871: 54, 87, 163, 183, 185; 1872: 9, 107; 1875: 228; 1877: 137.- Zeller 1875: 348.- Frey and Boll 1878: 275.- Riley 1891: 109, No. 5889.- Busck 1903: 189.- Dyar 1902 [1903]: 551, No 6267.- Braun 1908: 291.- Meyrick 1912a: 7; 1912b: 32.- Braun 1914: 110.- Forbes 1923: 192.- McDunnough 1939: 95, No. 9191.- Weaver and Dorsey 1965: 934; 1967: 178.
Phyllonorycter robiniella (Clemens).- Ely 1918: 59.- Davis 1983: 10.- Maier and Davis 1989: 15.- Leraut 1997: 96.- De Prins and De Prins 2005: 342.- De Prins and De Prins 2011.
Argyromiges pseudacaciella Fitch 1859: 836, No. 335.
Lithocolletis pseudacaciella (Fitch).- Riley, 1891: 109, No. 5889 (synonym of *Lithocolletis robiniella*).- Dyar 1902 [1903]: 551, No 6267.- Braun 1908: 291.- Meyrick 1912a: 7; 1912b: 32.- Ely 1918: 59.- Barnes and McDunnough 1917: 187, No. 7915.- McDunnough 1939: 95, No. 9191.- Davis 1983: 10.- Leraut 1997: 96.- De Prins and De Prins 2005: 342.- De Prins and De Prins 2011.

#### Diagnosis.

The overall appearance of this widespread eastern North American (and now well established European) species most closely resembles that of the more southwestern US species, *Macrosaccus neomexicanus*. The more abruptly constricted apical region of the valvae and the minute, longitudinally oriented striae and spicules of the corpus bursae readily distinguish it from *Macrosaccus neomexicanus*.

#### Adult

**Figures 2–9.**
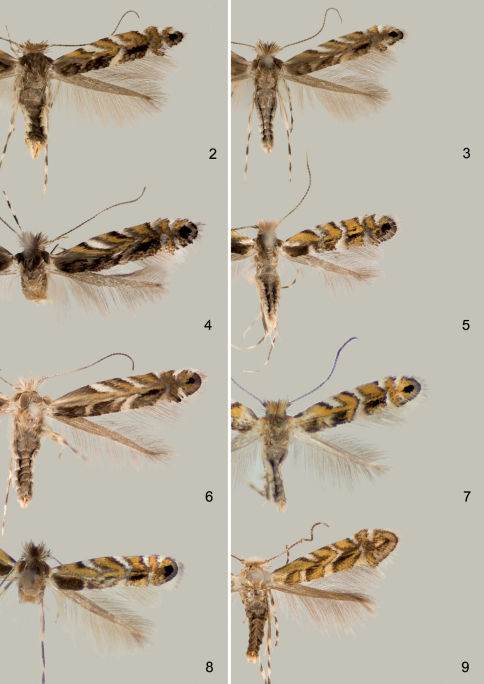
Adults **2–4.** *Macrosaccus robiniella*. **2**♂, USA: Maryland, (2.8 mm) **3** ♂, USA: Maryland, (3.0 mm) **4** BELGIUM: Antwerp, (3.0 mm) **5** *Macrosaccus morrisella*, ♂, USA: Maryland, (2.5 mm) **6** *Macrosaccus neomexicanus*, USA: Arizona, (3.2 mm) **7** *Macrosaccus uhlerella*, USA: Illinois, (2.5 mm) **8** *Macrosaccus uhlerella*, USA: Illinois, (3.0 mm) **9** *Macrosaccus gliricidius*, ♂, HONDURAS: Morazán, (2.4 mm). (Forewing lengths in parentheses).

(Figs 2–4). Forewing length 2.3–3.1 mm.

*Head:* Frons smooth, shiny white. Vertex extremely rough; vestiture consisting of a tuft of elongate, piliform, mostly dark brown, intermixed with white, scales. Labial palpus white. Antenna mostly dark fuscous dorsally for most its length, with dark area narrowing to a more slender dark streak toward basal 1/4–1/3 its length; antenna mostly white ventrally; apical segment entirely white.

*Thorax:* Dark brown dorsally, white ventrally; tegula dark brown, with pale grey to white suffusion anteriorly. Forewing pattern complex, costal half mostly light orange brown crossed by 4 equally spaced, white costal strigulae, each bordered basally, sometimes faintly, by black to dark grey and distally by light grey scales; basal 2 strigulae strongly oblique; a fifth, minute, white strigula sometimes arising from black apical spot before forewing apex. Basal third and dorsal half of forewing usually darker, mostly black to sometimes pale golden grey between strigulae; slender white streak from base of wing usually indistinct or absent; a greyish, oblique strigula often evident near base of wing which connects with a larger, more distinct greyish strigula from dorsal margin; dorsal margin also with 3, usually less distinct white strigulae approximately opposite to distal 3 white strigulae from costa; basal dorsal strigula usually contiguous with second costal strigula. Apex of forewing with a large black apical spot, which is rarely reduced; fringe mostly light grey. Hindwing, including fringe, uniformly grey. Foreleg mostly dark fuscous dorsally, white ventrally, with 2 white annuli around basal tarsomeres; midleg with 2 oblique bands of white dorsally over tibia; tarsomeres more broadly banded with white dorsally; hindleg mostly white with much of tibia pale fuscous dorsally, and with 3 broad, pale fuscous annuli dorsally over tarsomeres.

*Abdomen:* dark fuscous dorsally and white ventrally with greyish suffusion on anterior portion of segments 2–7 laterally and sometimes ventrally on A8.

*Male genitalia* (Figs 13, 14): Valva relatively simple, similar to *Macrosaccus morrisella* in form, gradually constricted before apex; apex rounded, densely setose; base of costa fused to moderately thickened, arched transtilla; transtilla with rounded knoblike lateral projections that extend anteriorly in repose (more caudally when valvae are spread widely apart); saccus a slender, elongate rod ~ 1.2× length of valva. Aedeagus very long and uniformly slender, ~ 2.1× length of valva.

*Female genitalia* (Figs 15–17): Ductus bursae long and slender, nearly half the length of elongate corpus bursae. Accessory bursae ~ 2/3 the length of corpus bursae, arising from anterior 1/3 of ductus bursae; with a smaller lateral pouch arising ~ midway along side of accessory bursae. Corpus bursae gradually broadening anteriorly, with faint longitudinal striae in wall which bear longitudinal rows of low, dentate ridges around anterior third of corpus bursae; walls of anterior end (distal 1/5) of corpus bursae entirely membranous.

**Figures 10–12.**
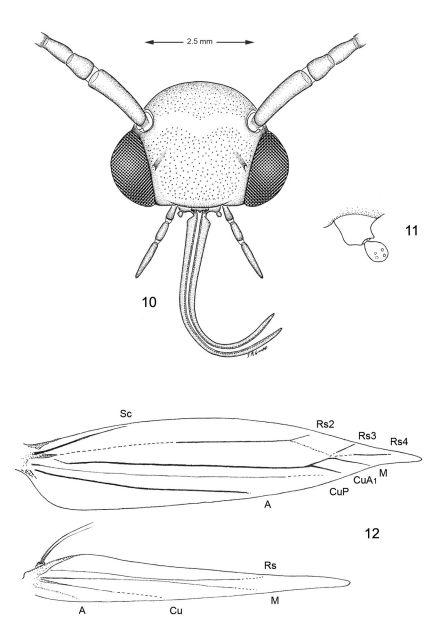
Adult morphology, *Macrosaccus robiniella*. **10** Head **11** Detail of left maxilla **12** Wing venation.

**Figures 13–17.**
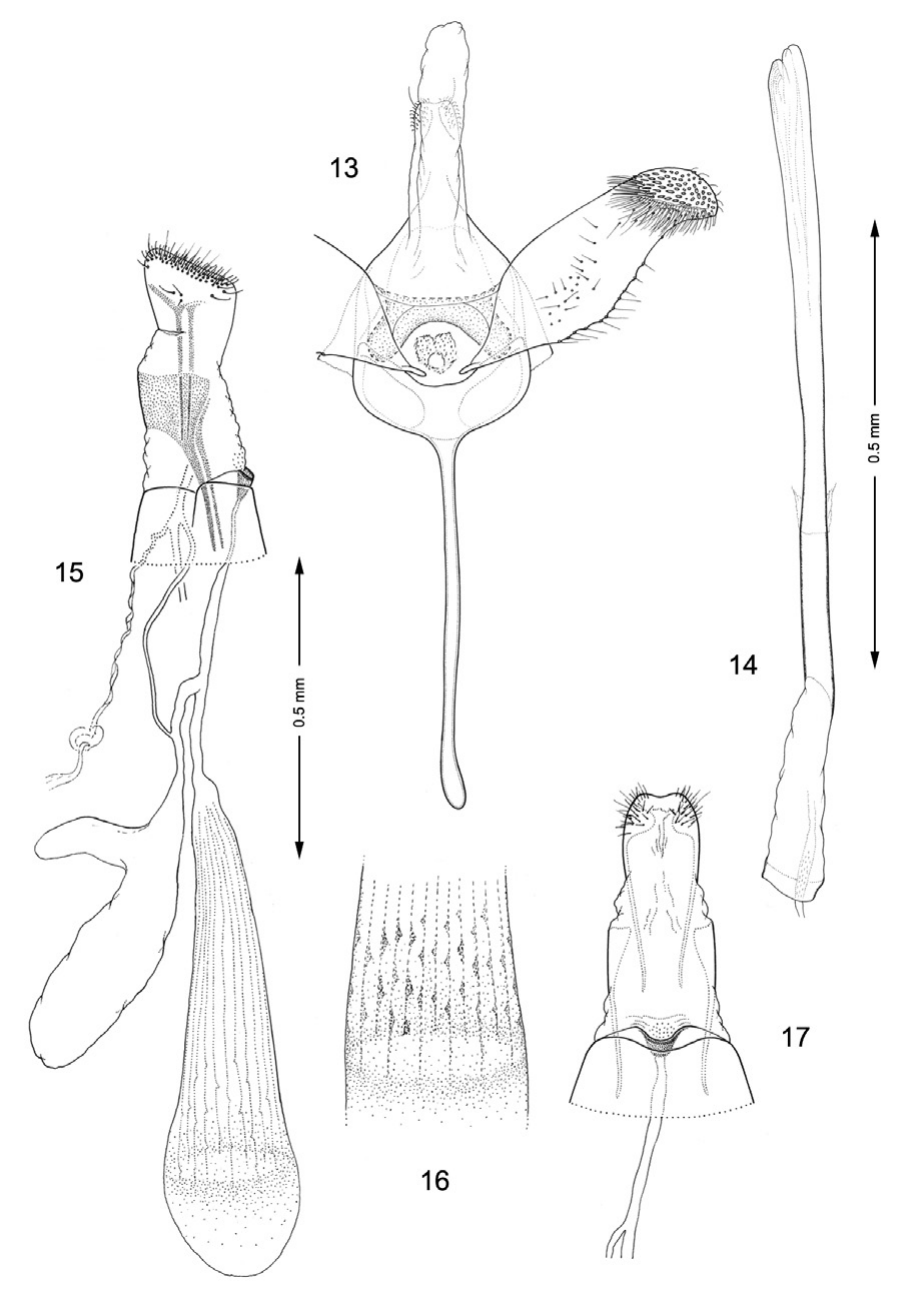
Genitalia**,** *Macrosaccus robiniella*. **13–14** Male. **13** Genital capsule, ventral view **14** Aedeagus. **15–17** Female. **15** Lateral view **16** Detail of signa within corpus bursae **17** Segments 7–10, ventral view.

#### Larva

(Figs 59–80, 90–96). Hypermetamorphic; five larval instars. Earliest instars (1–3) highly modified sapfeeders with strongly depressed bodies and reduced chaetotaxy; maximum length 3.7 mm, width (T1): 0.9 mm. Later instars (4 and 5) tissue feeders, with cylindrical bodies; maximum length: 4.7 mm, width: 0.7 mm; body colour pale green to white with notal plates and pinnacula smooth, reduced and unpigmented (indistinct).

#### Sap-feeding instars.

*Head*: Maximum width (third instar) 0.4 mm; greatly depressed, triangular. Most setae lost or reduced; 3 pairs of stemmata arranged in a lateral, anterior cluster on head. Labrum (Fig. 60) short and broad, bilobed, with 2 pairs of extremely reduced, peglike dorsal setae; anterior margin broadly concave, roughened, with 4–5 minute dentations along inner margin of lateral lobes. Mandibles broadly rounded, flattened, with 2 short cusps lateral to relatively large inner plate. Labium smooth, lateral margins subparallel; anterior margin shallowly notched at middle; spinneret absent. Maxillary and labial palpi absent. Hypopharynx broad, densely covered with minute spines along anterior margin; with margin slightly excavated at middle. Antenna 3-segmented, with short basiconic sensilla as shown (Fig. 62). *Body*: Setae generally reduced. Legs, prolegs, and crochets absent.

#### Tissue-feeding instars.

*Head*: Approximately round with full complement of mouthparts; brown; maximum width (fifth instar) 0.35 mm. Frons elongate, ~ 0.85× the distance to epicranial notch. Ecdysial line terminating near epicranial notch. Chaetotaxy (Figs 91–92) relatively complete; all three MD setae present, arising caudad to P1. P1 arising adjacent to ecdysial line. P2 reduced, arising slightly caudad to reduced L1. Setae AF1–2 absent. A2 arising near A3 in a line between P1 and A3. C1 and 2 reduced, closely adjacent. Four stemmata present. Antenna 3-segmented; first segment moderately long; sensilla as shown in Fig. 70. Labrum (Figs 68, 95) strongly bilobed with raised median portion on each lobe; M1 absent; numerous secondary spines visible from inner, ventral perimeter of labrum. Mandible (Figs 71, 96) with three large median cusps and one smaller median and two lateral cusps; mandibular setae variable (1–2) and located on anterior surface. Hypopharynx with dense, well developed dorsal spines. Maxilla as shown in Fig. 69. Spinneret a relatively short tube with a simple, rounded apex. Labial palpus with a relatively long basal segment bearing one short sensillum and a much shorter (~ 0.25× length of basal segment) apical bearing a single long apical sensillum ~ 2× length of apical segment. *Thorax*: Setae XD1 and 2 short, of equal lengths on prothorax (T1). SD1 elongate, immediately ventral to XD2; SD2 absent on T1, present on T2–3L group bisenose on T1–3. SV unisetose on T1–3. Legs (Fig. 76) relatively short but fully developed; coxae widely separated, with 4 coxal setae; pretarsal claw moderately curved. *Abdomen*: D and SD groups bisetose on A1–8, 10; unisetose on A9; L group bisetose on A1–5, unisetose on A6–10. Prolegs present on A3–5, 10; crochets of A3–5 consisting of 17–24 small hooks arranged in a uniordinal circle; anal proleg with crochets consisting of 15–18 small hooks arranged in a uniordinal semicircle opened caudally (Fig. 79). Anal plate with 4 pairs of setae.

#### Larval mine

(Figs 36–40). The mine begins as an elongate serpentine track (Fig. 37) which enlarges to an elongate-oval, whitish blotch Fig. 36, 38) located on one side of the midrib and usually on the under (abaxial) side of the leaflet. Eventually the mine becomes slightly tentiform due to the silk laid down by the later instar larvae.

#### Hosts.

(Table 1). Fabaceae: *Robinia pseudoacacia* L. (Clemens 1859: 320), *Robinia viscosa* Vent. (Chambers 1878: 111), *Robinia hispida* L. (Chambers 1878: 111; Needham et al. 1928: 288). The primary host, *Robinia pseudoacacia*, is believed once to have occurred primarily in two regions within the United States – one centered in the Appalachian Mountains from central Pennsylvania to northern Georgia and Alabama, and the other in the Ozark Plateau of southern Missouri, eastern Arkansas to eastern Oklahoma. This tree has since spread over much of the continental United States, portions of northeastern Canada, and parts of South America, Europe, Asia, Africa, and Australia (Stone 2009).

#### Life history

(Figs 36–40). The egg of *Macrosaccus robiniella* is deposited externally usually some distance from the leaf edge or midrib. Five larval instars have been observed by counting head capsules within mines in North America and Belgium. Kasch and Nicolai (2002) reported up to six instars based on head capsule measurements in Germany. The larvae typically form elongate-oval, whitish blotch mines on usually the under (abaxial) side of the leaflets. Upon eclosion, the apodal, prognathous sap-feeding larva enters the leaf and begins a slender, subepidermal, serpentine mine (Fig. 37). Eventually the mine is expanded into an oval blotch (Fig. 38) which usually encompasses and obliterates the previous serpentine mine. As is true for the larvae of *Phyllonorycter* (Davis and Deschka 2001), the last sap-feeding instar probably begins expanding the mine laterally. Initiation of the tissue-feeding instar is indicated by deeper feeding into the spongy and palisade tissue layers of the leaflet as the larva begins to ingest solid tissue. The resulting injury becomes visible from the opposite leaf surface, particularly in the underside mines, as dense, whitish punctures. As the tissue-feeding larva matures, it begins to lay down silken strands across the inner surface of the mine causing the leaflet to roll inwards and the mine to become tentiform (Fig. 39). Pupation occurs inside a silken cocoon (Fig. 40) within the mine without any precut exit opening. Especially during heavy infestations, the mines of adjacent larvae may coalesce resulting in multiple pupal cocoons. The phenology of this species has not been accurately determined over its range within North America. Normally two to three generations per year have been reported in Europe, which can occasionally reach as many as four (Nicolai 2005).

Braun (1908) noted that the mines could occur on both leaf surfaces. Weaver and Dorsey (1967) described the larval mining behaviour of *Macrosaccus robiniella* in great detail and observed several differences between the upper side mines, which reportedly were more common at higher elevations (~ 760 m), and the under side mines. The latter were found most frequently at elevations of ~ 270 m at their West Virginia study sites. Some of the distinctions they observed were that upper side mines occurred usually more basal on the leaflet and often extended across the midrib, with the larval frass concentrated more basally within the mine. Under side mines are situated less basally and usually restricted to one side of the midrib, with frass scattered more uniformly throughout the mine. Only a single, somewhat loosely woven cocoon was observed in the upper side mines, compared to as many as three, densely woven cocoons in the lower mines. DRD compared males reared from the upper and lower side mines and found no significant morphological differences (Weaver and Dorsey 1967). A search for the Weaver specimens in the collections of the University of West Virginia at Morgantown yielded no material associated with the upper side mines from the higher elevation sites (~ 760 m). Hopefully specimens from the higher elevation, upper side mines can be collected in order to examine their genetic distances.

In addition to Hymenoptera parasitoids, other Lepidoptera larvae have been noted within the mines of *Macrosaccus robiniella* (Weaver and Dorsey 1967). These were observed to alter the appearance of the mine by removing all mesophyll and largely destroying the frass pattern created by *Macrosaccus robiniella*. Packard (1890) identifies a species of Gelechiidae, *Filatima pseudacaciella* (Chambers), which sometimes feeds within the mine in addition to feeding externally.

#### Natural enemies.

(Table 4). Fifty seven species (including two unidentified) ofHymenoptera, the great majority of which are members of Eulophidae (Noyes 2010), have been reported as parasitoids of *Macrosaccus robiniella* in Europe and North America. Weaver and Dorsey (1967) also list two species of predators in the families Reduviidae and Vespidae that preyed on *Macrosaccus robiniella*

#### Pupa

(Figs 82–89, 97, 98). Maximum length 3.6 mm; width 0.9 mm. Vertex with frontal process (cocoon cutter) relatively short, broadly triangular, acute (Figs 81, 82). Forewing extending to anterior margin of A6; antenna slightly longer to middle of A6; hindleg extending to A7. Abdomen mostly covered dorsally and ventrally with dense, minute spines; dorsum of A2–7 with a single anterior row of short, stout spines (Figs 83, 98); caudal half of sternum 7 with a transverse ridge (accessory cremaster) bearing ~ 18–21 mostly longitudinal rows of short, blunt spines (Figs 84, 85). Cremaster of A10 greatly reduced, nearly absent, consisting of 1–2 pairs of minute tergal spines.

**Table 4.** Parasitoids of *Macrosaccus robiniella*.

Parasitoid name	Family	Country	Reference
*Achrysocharoides cilla* (Walker, 1839)	Eulophidae	Hungary	Csóka et al. 2009: 407
*Achrysocharoides gahani* (Miller, 1962)	Eulophidae	Italy	Navone 2003: 79
*Achrysocharoides gahani* (Miller, 1962)	Eulophidae	Switzerland	Girardoz et al. 2007: 606
*Achrysocharoides robiniae* Hansson & Shevtsova, 2010	Eulophidae	Austria	Hansson and Shevtsova 2010: 34
*Achrysocharoides robiniae* Hansson & Shevtsova, 2010	Eulophidae	Germany	Hansson and Shevtsova 2010: 34
*Achrysocharoides robiniae* Hansson & Shevtsova, 2010	Eulophidae	Hungary	Hansson and Shevtsova 2010: 34
*Achrysocharoides robiniae* Hansson & Shevtsova, 2010	Eulophidae	Italy	Hansson and Shevtsova 2010: 34
*Achrysocharoides robiniae* Hansson & Shevtsova, 2010	Eulophidae	U.S.A.	Hansson and Shevtsova 2010: 34
*Achrysocharoides robinicolus* Hansson & Shevtsova, 2010	Eulophidae	U.S.A.	Hansson and Shevtsova 2010: 34
*Ageniaspis testaceipes* (Ratzeburg, 1848)	Encyrtidae	Hungary	Csóka et al. 2009: 407
*Apanteles nanus* Reinhard, 1880	Braconidae	Italy	Bolchi Serini 1990: 142
*Astichus trifasciatipennis* (Girault, 1913)	Eulophidae	Italy	Noyes 2010: Internet
*Baryscapus nigroviolaceus* (Nees, 1834)	Eulophidae	Czech Republic	Girardoz et al. 2007: 608
*Baryscapus nigroviolaceus* (Nees, 1834)	Eulophidae	Hungary	Csóka et al. 2009: 407
*Baryscapus nigroviolaceus* (Nees, 1834)	Eulophidae	Italy	Gibogini et al. 1996: 16
*Baryscapus nigroviolaceus* (Nees, 1834)	Eulophidae	Switzerland	Girardoz et al. 2007: 606
*Chrysocharis laomedon* (Walker, 1839)	Eulophidae	Italy	Gibogini et al. 1996: 16
*Chrysocharis nephereus* (Walker, 1839)	Eulophidae	Switzerland	Whitebread 1990: 349
*Chrysocharis pentheus* (Walker, 1839)	Eulophidae	Hungary	Csóka et al. 2009: 407
*Chrysocharis pentheus* (Walker, 1839)	Eulophidae	Switzerland	Girardoz et al. 2007: 606
*Cirrospilus elegantissimus* Westwood, 1832	Eulophidae	Italy	Gibogini et al. 1996: 16
*Cirrospilus lyncus* Walker, 1838	Eulophidae	Hungary	Csóka et al. 2009: 407
*Cirrospilus talitzkii* Bouček, 1961	Eulophidae	Hungary	Csóka et al. 2009: 407
*Cirrospilus variegatus* (Masi, 1907)	Eulophidae	Italy	Gibogini et al. 1996: 16
*Cirrospilus viticola* (Rondani, 1877)	Eulophidae	Hungary	Csóka et al. 2009: 407
*Cirrospilus viticola* (Rondani, 1877)	Eulophidae	Italy	Gibogini et al. 1996: 16
*Closterocerus cinctipennis* Ashmead, 1888	Eulophidae	U.S.A.	Weaver and Dorsey 1965: 934
*Closterocerus* sp.	Eulophidae	Czech Republic	Girardoz et al. 2007: 608
*Closterocerus trifasciatus* Westwood, 1833	Eulophidae	Hungary	Csóka et al. 2009: 407
*Closterocerus trifasciatus* Westwood, 1833	Eulophidae	Italy	Bolchi Serini 1990: 143
*Colastes braconius* Haliday, 1833	Braconidae	Italy	Bolchi Serini 1990: 142
*Colastes braconius* Haliday, 1833	Braconidae	Switzerland	Whitebread 1990: 349
*Elachertus inunctus* Nees, 1834	Eulophidae	Italy	Zhu and Huang 2001: 343
*Eupelmus urozonus* Dalman, 1820	Eupelmidae	Hungary	Csóka et al. 2009: 407
*Hockeria unicolor* Walker, 1834	Chalcididae	Italy	Gibogini et al. 1996: 16
*Horismenus fraternus* (Fitch, 1856)	Eulophidae	U.S.A.	Weaver and Dorsey 1965: 934
*Mesochorus* sp.	Ichneumonidae	USA	Weaver and Dorsey 1967: 180
*Minotetrastichus frontalis* (Nees, 1834)	Eulophidae	Czech Republic	Girardoz et al. 2007: 608
*Minotetrastichus frontalis* (Nees, 1834)	Eulophidae	Hungary	Csóka et al. 2009: 407
*Minotetrastichus frontalis* (Nees, 1834)	Eulophidae	Italy	Bolchi Serini et al. 1990: 143
*Minotetrastichus frontalis* (Nees, 1834)	Eulophidae	Switzerland	Whitebread 1990: 349
*Necremnus hungaricus* (Erdös, 1951)	Eulophidae	Hungary	Csóka et al. 2009: 407
*Neochrysocharis formosus* (Westwood, 1833)	Eulophidae	Hungary	Csóka et al. 2009: 407
*Pediobius bucculatricis* (Gahan, 1927)	Eulophidae	Canada	Peck 1985: 677
*Pediobius liocephalatus* Peck, 1985	Eulophidae	Canada	Peck 1985: 675
*Pediobius saulius* (Walker, 1839)	Eulophidae	Hungary	Csóka et al. 2009: 407
*Pediobius saulius* (Walker, 1839)	Eulophidae	Italy	Gibogini et al. 1996: 16
*Pholetesor circumscriptus* Nees, 1834	Braconidae	Italy	Bolchi Serini 1990: 142
*Pholetesor nanus* (Reinhard, 1880)	Braconidae	Czech Republic	Girardoz et al. 2007: 608
*Pholetesor nanus* (Reinhard, 1880)	Braconidae	Hungary	Csóka et al. 2009: 407
*Pholetesor nanus* (Reinhard, 1880)	Braconidae	Italy	Bolchi Serini 1990: 142
*Pholetesor nanus* (Reinhard, 1880)	Braconidae	Switzerland	Girardoz et al. 2007: 606
*Pholetesor ornigis* Weed, 1887	Braconidae	U.S.A.	Weaver and Dorsey 1965: 934
*Pnigalio agraules* (Walker, 1839)	Eulophidae	Switzerland	Girardoz et al. 2007: 606
*Pnigalio pectinicornis* (Linnaeus, 1758)	Eulophidae	Hungary	Csóka et al. 2009: 407
*Pnigalio pectinicornis* (Linnaeus, 1758)	Eulophidae	Italy	Bolchi Serini 1990: 143
*Pnigalio pectinicornis* (Linnaeus, 1758)	Eulophidae	Switzerland	Girardoz et al. 2007: 606
*Pnigalio soemius* (Walker, 1839)	Eulophidae	Hungary	Csóka et al. 2009: 407
*Pnigalio soemius* (Walker, 1839)	Eulophidae	Italy	Bolchi Serini 1990: 143
*Pteromalus chrysos* Walker, 1836	Pteromalidae	Italy	Gibogini et al. 1996: 16
*Pteromalus* sp.	Pteromalidae	Czech Republic	Girardoz et al. 2007: 608
*Sympiesis acalle* (Walker, 1848)	Eulophidae	Hungary	Szabóky and Csóka 1997: 570
*Sympiesis acalle* (Walker, 1848)	Eulophidae	Italy	Bolchi Serini 1990: 143
*Sympiesis dolichogaster* Ashmead, 1888	Eulophidae	Switzerland	Girardoz et al. 2007: 607
*Sympiesis gordius* (Walker, 1839)	Eulophidae	Hungary	Csóka et al. 2009: 407
*Sympiesis gordius* (Walker, 1839)	Eulophidae	U.S.A	Weaver and Dorsey 1965: 934
*Sympiesis marylandensis* Girault, 1917	Eulophidae	U.S.A.	Maier 1988: 731
*Sympiesis sericeicornis* (Nees, 1834)	Eulophidae	Czech Republic	Girardoz et al. 2007: 608
*Sympiesis sericeicornis* (Nees, 1834)	Eulophidae	Hungary	Csóka et al. 2009: 407
*Sympiesis sericeicornis* (Nees, 1834)	Eulophidae	Italy	Bolchi Serini 1990: 143
*Sympiesis sericeicornis* (Nees, 1834)	Eulophidae	Switzerland	Girardoz et al. 2007: 607
*Sympiesis sericeicornis* (Nees, 1834)	Eulophidae	U.S.A.	Weaver and Dorsey 1965: 934

**Figures 18–22.**
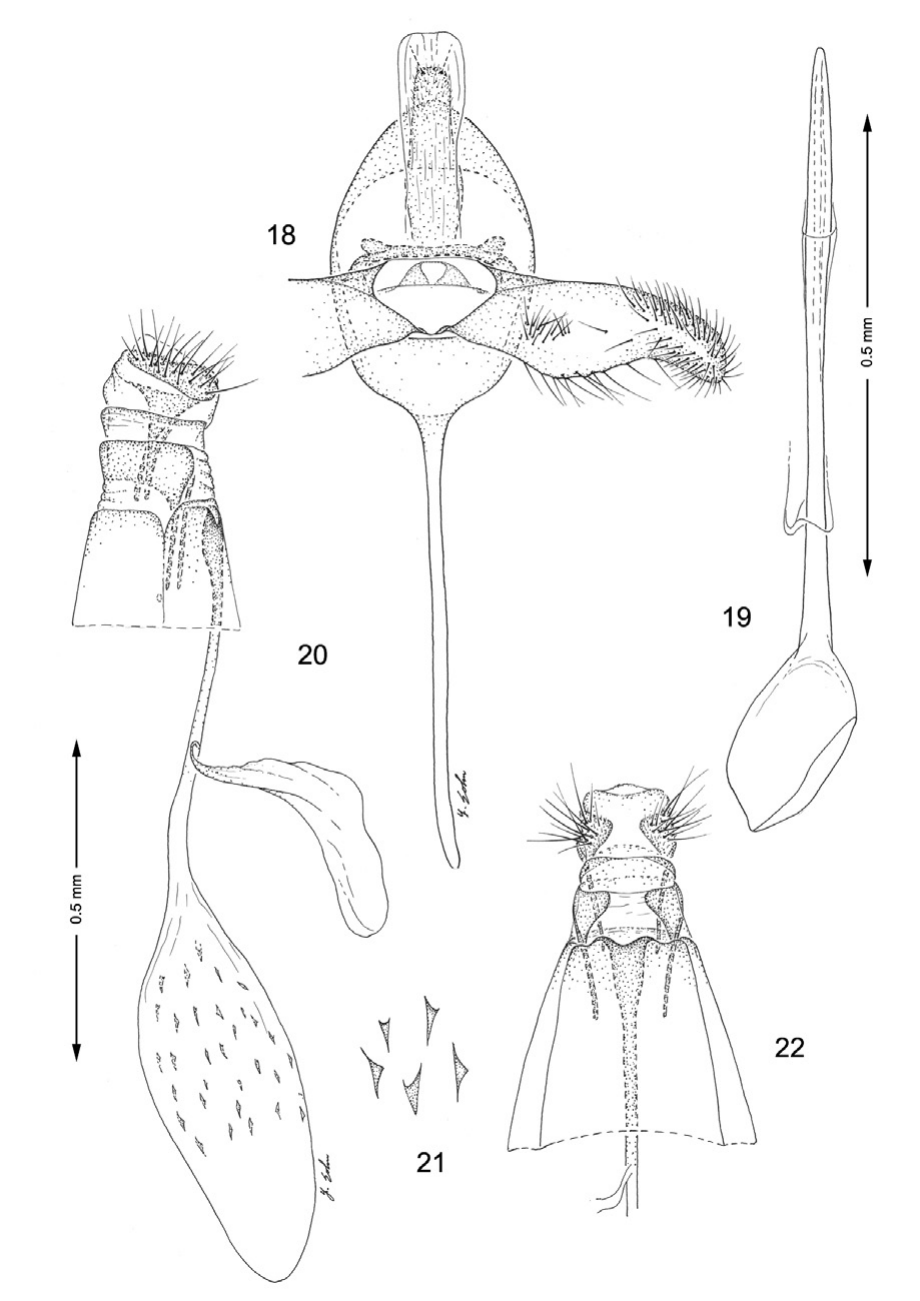
Genitalia**,** *Macrosaccus morrisella*. **18–19** Male. **18** Genital capsule, ventral view **19** Aedeagus **20–22** Female. **20** Lateral view **21** Detail of signa within corpus bursae **22** Segments 7–10, ventral view.

#### Types.

*Lithocolletis robiniella* Clemens: Lectotype ♀ (present designation): “14”; “*Lithocolletis robiniella* Clemens, Type ! A.B. 1902; Type 7505 *Lithocolletis robiniella* B. Clemens”; “Lectotype ♀ by D. R. Davis”, (ANSP). [The abdomen, right forewing, and distal part of right hindwing are missing].

Paralectotypes 3 ♂ and 1 specimen without abdomen “Syntype”, “*Lithocolletis robiniella* Clem. 1/4”, “Clemens det. ex Clemens coll.”, “Stainton coll. Brit. Mus. 1893–134”; same labels except nrs. 2/4, 3/4 and 4/4. The specimen with nr. 4/4 carries an extra label in Stainton’s handwriting: “*Lithocolletis robiniella* Clemens, Proc. N. S. Phil. 1859 p. 319, n.s. unlike any European species”, (BMNH).

*Argyromiges pseudacaciella* Fitch: Lectotype ♀ (present designation): “*Argyromiges Pseudacaciella*; Type No. 514 U.S.N.M”; “Lectotype ♀ by D. R. Davis.” (USNM).

#### Material examined.

BELGIUM: Province of Antwerp: Postel: 15 ♂, 23 ♀, 7 Sep 2009, em. 15–22 Sep 2009, J. and W. De Prins, leafmine on *Robinia pseudoacacia*, USNM slides 34257, 34258, 34263, DNA/BOLD ID RDOPO090-09, GenBank GU669590, DNA/BOLD ID RDOPO091-09, GenBank GU669591, (USNM). CANADA: ONTARIO: Ancaster: 1 ♂, 24 Jul 1964, T. N. Freeman, Host: *Robinia pseudoacacia*, 64–20, (CNC). Bobcaygeon: 1 ♂, 23 Jul 1932, J. McDunnough, reared on *Robinia*, (CNC). Ottawa: 1 ♂, 26 Aug 1955, G. G. Lewis, Host: B. locust, 55–8, (CNC). Walsh: 1 ♂, 23 Sep 1966, T. N. Freeman, Host: Bl. Locust, (CNC). UNITED STATES: DISTRICT OF COLUMBIA: 1 ♀, 7 Jul 1879, Host: *Robinia*, V.T. Chambers, (USNM); 3 ♂, 2 ♀, 9 Aug 1898, (USNM); 1 ♀, 24 Aug 1899 (USNM); 4 UNK, 18 Sep 1899, Host: *Robinia*, (USNM)*;* Rock Creek Park: 1 ♂, 22 May 1984, W. E. Steiner, (USNM). ILLINOIS: Adams Co: Quincy: 3 ♂, 3 ♀, 15–21 Feb 1948; 2 ♂, 2 ♀, 6 Apr 1947; J. P. Nielson (INHS). Coles Co: Fox Ridge State Park: 1 ♂, 1 Jun 1991, em. 9 Jun, 1991; 1 ♀, 29 Jun 1991, em. 5 Jul 1991; 3 ♂, 1 ♀, 14 Jul 1991, em. 16–20 Jul 1991, T. Harrison, leafmine on *Robinia pseudoacacia*, (INHS); 1 ♂, 7 Jun 1991, at UV light T. Harrison, (INHS). Putnam Co: 1 ♀, 15 Apr 1996; 1 ♂, 24 Apr 1963; 1 ♂, 29 Apr 1969; 1 ♂, 2 Jun 1966; 1 ♀, 11 Jul 1959; 1 ♀, 3 Sep 1951, M. O. Glenn, (INHS); 1 ♀, 3 May 1953; 1 ♂, 1 ♀, 10 May 1953; 1 ♂, 26 Aug 1961; 2 ♂, 10 Sep 1948; 2 ♂, 10 Oct 1948, M. O. Glenn, reared from *Robinia pseudoacacia*, (INHS). Vermilion Co: Kickapoo State Recreation Area: 1 ♂, 13 Jun 1991, em. 14 Jun 1991, T. Harrison, leafmine on *Robinia pseudoacacia*, (INHS). KENTUCKY: Fayette Co: Lexington: 1 ♂, 6–13 Oct 1975, malaise trap, (USNM). MARYLAND: Garret Co: Deep Creek. Lake State Park: 15 ♂, 12 ♀, 16 Sep 1990, em. 23 Sep – 7 Oct 1990, D. and S. Davis, DRD 821, Host: *Robinia pseudoacacia* L. USNM slides 33282, 30903, 30895, 30894, DNA/BOLD ID RDOPO088-09, GenBank GU669592, DNA/BOLD ID RDOPO089-09, GenBank GU669593, (USNM). Montgomery Co: Fort Washington, vicinity Henson Creek: 1 ♂, 19 Sep 1990, em. 13 Oct 1993, D. Davis, DRD 1376, Host: *Robinia pseudoacacia* L., (USNM). MASSACHUSETTS: Essex Co: Beverly: 1 ♂, 2/69, Burgess, (BMNH). MICHIGAN: Clinton Co: T6N-R1W S10: 3 ♂, 2 Oct 1997, em. 9–13 Oct 1997, R. J. Priest, Host: *Robinia pseudoacacia* L., (USNM). Wayne Co: Detroit: 1 ♂, 2 ♀, 20 Nov 1995, T. Wallenmeier, (USNM). MISSOURI: Boone Co: Columbia: 1 ♂, 25 Nov 1995, slide USNM 17047, 1 ♀, 14 Dec 1969, W.S. Craig, under bark of sycamore, (USNM). NEW HAMPSHIRE: Cumberland Co: Hampton: 1 ♀, 16 Feb 1906, S.A. Shaw, (USNM). NEW JERSEY: Burlingnton Co: Moorestown: 2 ♂, 22 Aug 1902, W.D. Kearfott, Host: Locust, (USNM). NEW YORK: Specific locality unknown: 1 ♀, lectotype, *Argyromiges pseudacaciella* Fitch, (USNM). Clinton Co: Peru: 2 ♂, 2–18 May 1977, R. Weires, caught in pheromone trap, slide USNM 20912, (USNM). Essex Co: Crown Point: 2 ♂, 4–20 May 1977, 1 ♂, 20 May-17 Jun 1977, R. Weires, caught in pheromone trap, slide USNM 20910, (USNM). Livingston Co: Letchworth State Park: 12 ♂, 8 ♀, 21–22 Jun 1986, E. R. Hoebeke, reared from mines of *Robinia pseudoacacia*, (CU). Thompkins Co: Ithaca: 1 ♂, 15 Feb; 1 ♂, 1 ♀, 8 Apr 1945, Renwick, (CU). NORTH CAROLINA: Macon Co: Highlands, 3865’: 6 ♂, 5 ♀, 1–24 Aug 1958, R. W. Hodges, (CU); 11 ♂, 5 ♀, 27 Jul-25 Aug 1958, R.W. Hodges, (USNM). OHIO: Hamilton Co: Cincinnati: 1 ♂, 29 Apr 1905, 6806, (CNC); 1 ♀, 29 Apr 1903, 7 ♂, 4 May 1904, 1 ♂, 23 July 1903, 1 ♂, 1 ♀, 25–27 Sep 1902, 1 ♂, 1 ♀, 30 Sep 1911, slide USNM 97837, 2 ♀, 10–22 Oct 1903, 3 ♂, 15–20 Nov 1903, Annette F. Braun, (USNM). PENNSYLVANIA: Specific locality unknown: 1 ♀, lectotype, *Lithocolletis robiniella* Clemens, (ANSP). Allegheny Co: Oak Station: 1 ♂, 1 ♀, 1 Oct 1910, Fred Marloff, (CU); 1 ♀, 10 Apr 1910, 3 ♂, 5 ♀, 3–22 May 1910, 1 ♀, 12 Jun 1908, Fred Marloff, (USNM). Erie Co: Girard: 3 ♂, 1 ♀, 9 Oct 1920, reared from black locust, (CU). Franklin Co: Mont Alto: 1 ♀, 5 Oct 1971, reared Black locust seedling, slide USNM 17166, (USNM). Indiana Co: Strongstown: 1 ♀, 23 Sep 1971, reared Black locust seedling, (USNM). Monroe Co: Sciota: 1 ♂, 21 Jul 1965, T. N. Freeman, Host: *Robinia pseudoacacia*, 65–27, (CNC). SOUTH CAROLINA: Oconee Co: Cherry Hill Rec. Area, Rt.107, 2000’ [610m]: 2 ♂, 1 ♀, 11 Aug 1958, R.W. Hodges, (CU); 3 ♂, 11 Aug 1958, R.W. Hodges, slide USNM 17017, (USNM). TEXAS: 1 ♀, Boll, (USNM). VIRGINIA: Arlington Co: Rosslyn: 1 ♂, A. Busck, underside mine on Hog peanut, (USNM). Madison Co: Shenandoah Nat. Park, Skyline: 3 ♀, 12 Aug 1972, E. Jäckh, Host: *Robinia pseudoacacia* L., (USNM). WEST VIRGINIA: Marion Co: Morgantown: 5 ♂, 3 ♀, [no date], Host: *Robinia*, slide USNM 97835, (USNM). WISCONSIN: Dane Co: Madison: 1 ♂, 1 ♀, 24 Aug 1958, L. J. Bayer, Host: *Robinia*, (USNM).

#### Distribution.

*Macrosaccus robiniella* occurs naturally over much of eastern North America from Ontario, Canada south to South Carolina and west to Missouri and Texas. *Macrosaccus robiniella* was first reported in Europe in 1983, near Basel, Switzerland (Whitebread 1990: 344) and has since spread through 23 European countries (Table 5).

**Table 5.** Discovery and general distribution of *Macrosaccus robiniella* in Europe.

Country	First year of occurrence	Reference to the first record
Albania	not recorded	Lopez-Vaamonde et al. 2010: 645
Austria	1991	Huemer et al. 1992: 199
Belgium	2001	De Prins and Groenen 2001: 159
Bosnia and Herzegovina	1999	Dimić et al. 2000: 7
Bulgaria	2001	Tomov 2003: 105
Croatia	2000, unpublished observations, Aleš Laštůvka & Hana Šefrová, pers. comm.	Lopez-Vaamonde et al. 2010: 645
Czech Republic	1992	Laštůvka et al. 1993: 31
Denmark	2003	Buhl et al. 2005: 79
France	1984	Whitebread and Joos 1986: 117
Germany	1988–1989	Whitebread 1990: 345
Hungary	1992	Szabóky and Csóka 1997: 569
Italy	1988	Bolchi Serini and Trematerra 1989: 193
Lithuania	2007	Noreika 2008: 35
Moldova(Pridnestrovje)	2006	Antyukhova 2007: 65
Netherlands	1999	De Prins and Groenen 2001: 160
Poland	1999 from Šefrová 2002: 10	Buszko and Novacki 2000: 25
Romania	2002	Neţoiu 2003: 154
Serbia	1998	Dimić et al. 1999: 34
Slovakia	1992	Buszko 1996: 53
Slovenia	1994	Seljak 1995: 78
Spain	2001	Olivella 2002: 35
Switzerland	1983	Whitebread and Joos 1986: 117
Ukraine	2002	Bidzilya and Budashkin 2004: 61

#### Remarks.

Thesynonymousnames *Lithocolletis robiniella* Clemens and *Argyromiges pseudacaciella* Fitch were both published in 1859. The month of publication for *robiniella* is clearly indicated as November in the Proceedings of the Academy of Natural Sciences of Philadelphia for that year. The month of publication for *pseudacaciella* Fitch cannot be determined as precisely. With the assistance of Tim McCabe of the New York State Museum, we were able to resolve an approximate date of printing for the Fifth report of Fitch’s Report on the noxious, beneficial and other insects of the state of New York (Fitch 1859), but we were not able to determine the actual distribution date. From such dated sections of that Report, particularly a “Notice “ to the farmers of New York, McCabe deduced that the Fifth Report most likely was printed in March, 1859. Attempts to locate receivership stamps for this report in various libraries to determine an approximate distribution date have been unsuccessful.

Thus, available evidence now suggests that *pseudacaciella* Fitch preceded the publication of *robiniella* Clemens by a few months. Because it is known that (1) Riley (1891) first treated *pseudacaciella* as a junior synonym of *robiniella* and no subsequent author has considered it otherwise, and (2) that the name *robiniella* has been recognized as the valid name for this taxon in more than 25 publications (141 publications using *robiniella* as the valid name are actually known) by more than 10 authors, this name must be preserved as the valid name for this taxon in accordance with the provisions of article 23.9.1 of the International Code of Zoological Nomenclature (ICZN 1999).

Neither the type locality nor the number of specimens examined were provided by Fitch for *Argyromiges pseudacaciella*. The same is true for the other two species of Gracillariidae Fitch proposed in 1859, *Argyromiges morrisella*, and *Argyromiges uhlerella*. Because it is believed that most of Fitch’s collecting occurred within the vicinity of his “bug house” (still standing and now a historical site) in Salem, New York, it is likely that the type locality for all three species may be from this general area (McCabe, in litt.).

### Macrosaccus morrisella

(Fitch) comb. n.

http://species-id.net/wiki/Macrosaccus_morrisella

Figs 1 5 41–44 18–22 Tables 1 2

Argyromiges morrisella Fitch 1859: 838, No. 336.
Lithocolletis morrisella (Fitch).- Chambers 1871: 183.- Walsingham 1889: 52.- Riley 1891: 109, No. 5874.- Dyar 1902 [1903]: 551, No. 6269.- Braun 1908: 291.- Meyrick 1912a: 7; 1912b: 33.- Braun 1914: 110.- McDunnough 1939: 95, No. 9189.
Phyllonorycter morrisella (Fitch).- Ely 1918: 58.- Davis 1983: 10.- Maier and Davis 1989: 15.- De Prins and De Prins 2005: 323.- De Prins and De Prins 2011.
Lithocolletis texanella Zeller 1875: 349.- Frey and Boll 1878: 275.- Walsingham 1889: 52 (synonym of *Phyllonorycter morrisella* (Fitch))- Riley 1891: 109, No. 5874.- Dyar 1902 [1903]: 551, No. 6269.- Braun 1908: 291.- Meyrick 1912a: 7; 1912b: 33.- Braun 1914: 110.- Ely 1918: 59.- Forbes 1923: 192.- McDunnough 1939: 95, No. 9189.- Davis 1983: 10.- Maier and Davis 1989: 15.- De Prins and De Prins 2005: 323.- De Prins and De Prins 2011.
Lithocolletis texana (Chambers 1877: 137) [misspelling].- Davis 1983: 10.
Lithocolletis amphicarpeaeella Chambers 1877: 137.- Riley 1891: 109, No. 5874 (synonym of *Phyllonorycter morrisella* (Fitch))- Dyar 1902 [1903]: 551, No. 6269.- Braun 1908: 291.- Meyrick 1912a: 7; 1912b: 33.- Ely 1918: 59.- McDunnough 1939: 95, No. 9189.- Davis 1983: 10.- De Prins and De Prins 2005: 323.- De Prins and De Prins 2011.
Lithocolletis amphicarpaeella Riley 1891: 109, no. 5874 [misspelling].- Braun 1908: 291.- Davis 1983: 10.

#### Diagnosis.

The forewing pattern of this species differs from that of *Macrosaccus robiniella* and *Macrosaccus neomexicanus* in possessing a more distinct basal white streak, in having the dorsal strigulae oriented less obliquely, and with the basal white dorsal stigula more pronounced, and from *Macrosaccus gliricidius* by the darker ground colour. The forewing pattern most resembles that of *Macrosaccus uhlerella* but differs in the more pronounced basal white streak which is absent or barely evident in *Macrosaccus uhlerella*. The male genitalia are most similar to that of *Macrosaccus robiniella*, particularly with regard to the more abruptly constricted apical third of the valva. The female genitalia differ from the latter in lacking the minute longitudinally oriented striae and spicules in the walls of the corpus bursae.

##### Adult

(Fig. 5). Forewing length 2.3–2.8 mm.

*Head*: Vestiture of head and antenna similar to *Macrosaccus robiniella*.

*Thorax*: Dark brown to fuscous dorsally, sometimes with a coppery to purplish luster; shiny white ventrally; tegula dark brown, with white suffusion anteriorly. Forewing pattern similar to *Macrosaccus robiniella* except basal two costal strigulae less oblique; a slender white, slightly oblique streak usually well developed extending distad from tegula at base of wing to sometimes as far as first dorsal strigula; 3 white dorsal strigulae usually present, but these oriented less obliquely than in *Macrosaccus robiniella*; basal strigula white, but sometimes obscure; median strigula connected to second costal strigula to form a narrow white fascia; black apical spot present as in *Macrosaccus robiniella*; cilia light grey to white. Hindwing, including fringe, uniformly grey. Legs similar to *Macrosaccus robiniella* in colour pattern.

*Abdomen:* Similar to *Macrosaccus robiniella*, dark fuscous dorsally and white ventrally with greyish suffusion on anterior portion of segments 2–7 laterally and sometimes ventrally on A8.

*Male genitalia* (Figs 18, 19): Similar to *Macrosaccus robiniella*, with valva gradually constricting before apex. Saccus a long, slender rod ~ 1.75× length of valva. Aedeagus long and slender, ~ 3.0× length of valva, with phallobase slightly more enlarged than in *Macrosaccus robiniella*.

*Female genitalia* (Figs 20–22): Ductus bursae long and slender, nearly equal to length of of elongate corpus bursae. Accessory bursae ~ 2/3 the length of corpus bursae, arising from near anterior 1/3 of ductus bursae. Corpus bursae elliptical, with series of small, scattered dentate spicules concentrated over caudal 2/3; longitudinal folds or striae not evident along walls; walls of anterior end (distal 1/3) of corpus bursae entirely membranous.

#### Larva and pupa.

Similar to that of *Macrosaccus robiniella*.

#### Larval mine.

(Figs 41–44) The mine begins as an elongate serpentine track on the under (abaxial) side of the leaflet. This enlarges to an elongate-oval, whitish blotch which eventually becomes strongly tentiform (Fig. 43).

#### Hosts.

(Table 1). Fabaceae: *Amphicarpa bracteata* (L.) Fernald, (=*Amphicarpa monoica* (L.) Nutt.,= *Falcata comosa* (L.) Kuntze, = *Amphicarpa comosa* (L.) Loudon), (Chambers 1878: 111; Walsingham 1889: 119; Ely 1917: 59). *Strophostyles leiosperma* (Torrey and A. Gray), (= *Strophostyles pauciflorus* (Bentham) S. Watson), new record. The primary host, *Amphicarpa bracteata*, is a low growing, trifoliate vine which occurs in damp woodlands widely from southern Canada and Montana to Texas and Florida. *Strophostyles leiosperma* is a climbing, trifoliate vine which occurs in drier habitats through the central United States from Arizona to Pennsylvania and Florida.

#### Types.

*Argyromiges morrisella* Fitch: Lectotype ♂, (present designation): “Type; *Argyromiges morrisella* Ft.; Figured by Miss A. Braun, Feb. 1908; Lectotype ♂, *Macrosaccuss morrisella* (Fitch) by D. R. Davis.” (USNM). [Abdomen is missing].

*Lithocolletis texanella* Zeller: Holotype ♂, “Type 1336”; “Dallas, Texas”, (MCZ). Paratype ♂, “Type”, “*Lithoc. Texanella* Z. B1/77”, “Dallas, Texas”, “Zeller Coll., Walsingham Collection 1910–427”, “*Lithocolletis morrisella* Fitch”, (BMNH).

*Lithocolletis amphicarpeaeella* Chambers: Lectotype ♂, (present designation): “Type, 1326”; “Kentucky [crossed out], Chambers”; “*Lithocolletis amphicarpeaeella* Cham.”; “Lectotype♂, *Lithocolletis amphicarpeaeella* Chambers, by D. R. Davis”, (MCZ); Paralectotype ♂, “N J, Chambers 1/77” “*Lith. Amphicarpeaeella* Nr. 3, Syntype, select KRT”, (BMNH).

Note: In the lower right corner of drawer Mi 4423, a label with the following handwritten text is present: “Nb. Chambers’ syntypes. The specimens bearing the serial numbers 1–68, and the specimens of *G. hermannella* var. *lingulacella* immediately following, were received by Stainton from Chambers. The numbers refer to a list made by Chambers; many of the specimens are likely to be syntypes. See Stainton foreign correspondence, letters nos. 94, 97 and 111 (by Chambers) and Stainton’s “translations” of Chambers’ awful handwriting, nos. 95, 99 and 96 respectively (the letters are out of chronological sequence). – KRT, 1980”.

#### Material examined.

CANADA: MANITOBA: 1 ♀, Aweme: 31 Aug 1931, Host: *Strophostyles pauciflora* (=*Strophostyles leiosperma*), R. M. White, (CNC). ONTARIO: Pt. Pelee: 1 ♀, 10 Oct 1967, T. N. Freeman, Host: Hog Ranot 63–46, (CNC). Simcoe: 1 ♂, 15 Sep 1955, T. N. Freeman, Host: *Amphicarpa monoica* (=*Amphicarpa bracteata*) 65–85, (CNC). Toronto: 2 ♂, 2 ♀, 5.22, Parish; 1 ♂, 6.22, Parish, (BMNH). QUEBEC: Fairy Lake: 1 ♂, 28 Aug 1955, Host: *Amphicarpa* 55–178, G. G. Lewis, (CNC). UNITED STATES: COLORADO [no specific locality provided]: 1 ♂, Type 1326, [Kentucky crossed out] Chambers, Lectotype *Lithocolletis amphicarpeaeella* Cham. ♂, (MCZ); 1 ♂, 1 ♀, Type 1326, [Kentucky crossed out] Chambers, Paralectotype *Lithocolletis amphicarpeaeella* Cham., (MCZ). CONNECTICUT: Hartford Co: Southington: 1 ♂, 3 Sep 1981, C. T. Meier, Host: *Amphicarpa bracteata*, Leaflet, (USNM). KENTUCKY: 1 ♀, Chamb., 132, (MCZ). ILLINOIS: Adams Co: Quincy: 1 ♀, 15 Feb 1948; 2 ♂, 3 ♀, 6–20 Apr 1947; J. P. Nielson, (INHS). Coles Co: Fox Ridge State Park: 1 ♀, 1 Jun 1991, em. 9 Jun 1991; 1 ♂, 1 ♀, 7 Jun 1991, em. 10 Jun 1991; 2 ♂, 3 ♀, 17 Jun 1991, em. 18–25 Jun 1991; 14 Jul 1991, em. 15 July 1991; 1 ♂, 2 ♀, 2 Aug 1992, em. 6–9 Aug 1992, T. Harrison, Host: *Amphicarpa bracteata*, (INHS). Putnam Co: 2 ♂, 1 ♀, 15 Jan 1941; 1 ♀, 8 Oct 1964; 2 ♂, 3 ♀, 13–30 Sep 1967; 4 ♂, 1 ♀, 2–8 Oct 1969, underside tentiform mine *Amphicarpa monoica*, M. O. Glenn, (INHS). MARYLAND: Montgomery Co: 2 mi. S. Laytonsville: 1 ♂, DOA 8 Oct 2009, D.R. Davis, Host: *Amphicarpa bracteata*, (USNM). Little Bennett Regional. Park: 3 ♂, 3 Aug 2002, em. 14 Aug 2002, D. R. and S. R. Davis, 3 ♂, 5 ♀, 6 Aug 2006, em. 10–16 Aug 2006 DNA/BOLD ID RDOPO081-09, GenBank GU669599, DNA/BOLD ID RDOPO083-09, GenBank GU669598 ; 2 ♂, 7 Aug 2010, em. 16–18 Aug 2010, D. R. and M. M. Davis, DRD slide 2664, Host: *Amphicarpa bracteata*, slides USNM 34180, 34181, 34182, DNA/BOLD ID 00715473, DNA/R50BOLD ID 00715475, (USNM). Plummers Island: 1 ♂, 5 Nov 1914, Shannon, (USNM). Prince Georges Co: Seton Belt Woods: 1 ♂, 11 Jul 1977, E. Jäckh, (USNM). MICHIGAN: Calhoun Co: 4 ♂, T1S-R6W Sec 15: 31 Aug 1996, em. 7–11 Sep 1996, lot RJP654.17-18, Host: *Amphicarpa bracteata*. Clinton Co: T05N-R01 R01W S24: 28 Aug 1991, em. 7–11 Sep 1996, R. J. Priest, lot RJP 932.6-7, Host: *Amphicarpa bracteata*, (USNM). NORTH CAROLINA: Macon Co: Highlands, 3865’: 1 ♂, 31 Jul 1958, R. W. Hodges, (USNM). NEW YORK: Thompkins Co: Ithaca, Six Mile Creek: 3 ♂, 1 ♀, 8 Apr 1945, (CU); 1 ♂, 10 Jul 1960, 1 ♂, 10 Jul 1960, R. W. Hodges, Host: *Amphicarpa monoica* (L.), (USNM). OHIO: Hamilton Co: Cincinnati: 1 ♂, em. 23 Aug 1908, Annette F. Braun, B361, (MCZ); 1 ♀, em. 16 Jul 1908, 1 ♂, em. 2 Aug 1908, 1 ♂, em. 23 Aug 1908, 1 ♂, em. 24 Aug 1908, 1 ♂, em. 25 Aug 1908, 1 ♂, 1 ♀, em. 2 Sep 1908, 1 ♂, 5 Sep 1918, slides USNM 30891, 30892, 97446, 97447, (USNM). TEXAS: 3 ♂, 1 ♀, Boll, (BMNH). Dallas Co: Dallas: 2 ♂, (holotype and paratype of *Lithocolletis texanella* Z.), type 1336, Boll, (MCZ); “*Lithoc Texanella* Z, B 1/77, type”, (BMNH); 2 ♀, (*Lithocolletis texanella* Z.), (BMNH); 3♂, 3♀ (*Lithocolletis texanella* Z.) in coll. Stainton (BMNH).

#### Distribution.

*Macrosaccus morrisella* occurs widespread across eastern North America from Manitoba and Ontario, Canada, south and west to Texas and Colorado (Chambers 1877).

#### Remarks.

Chambers in his 1877 description of *Lithocolletis amphicarpeaeella* expressed doubt if this was a new species or new variety (as he did on the same page and line for “*Lithocolletis amorphaeella* n.sp.? or var.?”). Their descriptions included long, detailed comparisons of the forewing patterns of these two new moths as well of *robiniella* and “*texana*” (misspelled). No locality for either name was mentioned except for Colorado in the title of the publication (Chambers 1877). Each of the three “type” specimens of *amphicarpeaeella* in the collections of the MCZ have the name “Kentucky” crossed out on the specimen label “Kentucky, Chambers”, and no mention of Colorado appears. A male specimen in best condition has been selected as lectotype.

The holotype specimen of *Lithocolletis texanella* Zeller upon examination was found to be a male and not a female as stated originally by Zeller (1875).

### Macrosaccus neomexicanus

Davis sp. n.

urn:lsid:zoobank.org:act:3FD42A3B-6E1B-4788-82F7-D9B2AF3D3354

http://species-id.net/wiki/Macrosaccus_neomexicanus

Figs 1 6 23–27 45–50 Tables 1 2

#### Diagnosis.

As discussed in the diagnosis of *Macrosaccus robiniella*, this species most resembles the former in general appearance. They differ in distribution, host preference, in genital morphology (see diagnoses of *Macrosaccus robiniella*), and possibly overall size, with the wingspan of *neomexicanus* being slightly larger.

#### Adult

(Fig. 6). Forewing length 2.7–3.5 mm.

*Head:* Vestiture of head and antenna similar to *Macrosaccus robiniella*; apical segment of antenna white to grey.

*Thorax:* Dark brown dorsally, with whitish suffusion anteriorly and laterally; white ventrally; tegula dark brown, with pale grey to white suffusion anteriorly. Forewing and hindwing patterns very similar to *Macrosaccus robiniella*. Vestiture of legs similar to *Macrosaccus robiniella*.

*Abdomen:* Similar to *Macrosaccus robiniella*, dark fuscous dorsally and white ventrally with greyish suffusion on anterior portion of segments 2–7 laterally and sometimes ventrally on A8.

**Figures 23–27.**
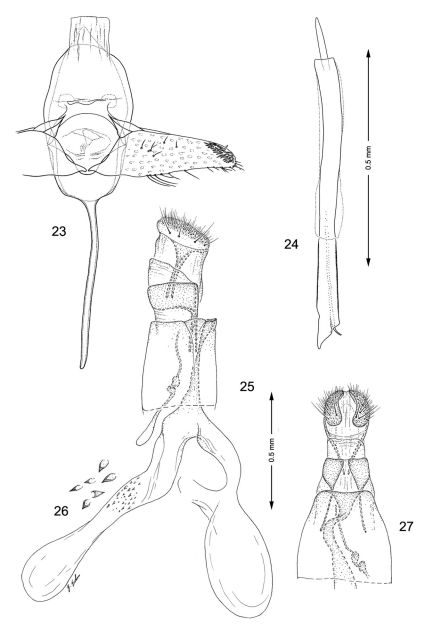
Genitalia**,** *Macrosaccus neomexicanus*. **23–24** Male. **23** Genital capsule, ventral view **24** Aedeagus **25–27** Female. **25** Lateral view **26** Detail of signa within corpus bursae **27** Segments 7–10, ventral view.

*Male genitalia* (Figs 23, 24): Valva relatively simple, gradually narrowing before apex without abrupt constriction; apex narrowly rounded, densely setose, particularly along costal margin. Saccus a slender, elongate rod ~ 1.3× length of valva. Aedeagus very long and uniformly slender, ~ 2.5× length of valva.

*Female genitalia* (Figs 25–27): Ductus bursae moderately long and slender, ~ 1/3 the length of elongate corpus bursae. Accessory bursae nearly as long as corpus bursae, arising from junction of ductus bursae and corpus bursae; with a smaller lateral pouch arising ~ midway along side of accessory bursae. Corpus bursae relatively slender, anterior end only slightly broader; a dense scattering of minute spicules encircling middle; remaining walls of corpus bursae entirely membranous.

#### Larva and pupa.

Similar to that of *Macrosaccus robiniella*.

#### Larval mine

(Figs 46–50). The mine begins as a relatively short, serpentine track which enlarges to an elongate-oval, whitish blotch located on the under (abaxial) side of the leaflet. As the larva develops and begins laying down silk, the mine becomes strongly tentiform, causing the upper (adaxial) surface to roll over (Figs 48, 49).

#### Host.

(Table 1). Fabaceae: *Robinia neomexicana* Gray. The host is a moderately small, spiny shrub growing to as high as 5 meters and usually forming dense thickets (Fig. 45). It occurs from California to Texas and north to Wyoming.

#### Life history

(Figs 46–50). Some collections of this species from southern Arizona have been from dense infestations. In such populations, oviposition tends to be concentrated on fewer available leaflets with as many as 45 short, initial serpentine mines observed on a single leaflet. These soon coalesce resulting in a single large blotch covering nearly the entire lower side of the leaflet (Fig. 47). Larval mortality is probably high under these conditions. One large composite mine opened contained 14 live and 11 dead, late instar larvae and no pupae. A maximum of 11 cocoons with pupae (5 on one side of the midrib and 6 on the other side) were found in one leaflet (DRD rearing lots 541, 541.1, Fig. 50).

#### Holotype.

**♂**: UNITED STATES: ARIZONA: Cochise Co: Carr Canyon: Huachuca Mts: 20 Sep 1985, em. 27 Sep 1985, HOST: *Robinia neomexicana*, R. S. Wielgus, digital image captured, (USNM).

#### Paratypes.

UNITED STATES: ARIZONA: Coconino Co: North Rim Grand Canyon: 1 ♀, 15–16 Aug 1978, em. 17 Aug – 6 Sep 1978, G. Deschka, HOST: *Robinia neomexicana*, (BMNH). Cochise Co: Carr Canyon: Huachuca Mts: 70 ♂, 57 ♀, 20 Sep 1985, em. 21 Sep - 2 Oct 1985, R.S.Wielgus, DRD541, HOST: *Robinia neomexicana*, slides USNM 28416, 30890, 34267, (USNM). NEW MEXICO: Catron Co: Rocky Canyon., Gila Nat. Forest: 1 ♂, 1969, em. 6 Aug 1969, D. & M. Davis, DRD642.2, Host: *Robinia neomexicana*, (USNM). Otero Co: Deerhead Campground, ca. 2 mi. S. Cloudcroft: 1 ♂, 1 ♀, 18–19 Jul 1969, em. 2 Aug 1969, D. & M. Davis, DRD 642, Host: *Robinia neomexicana*, DNA/BOLD ID RDOPO084-09, GenBank GU669596, DNA/BOLD ID RDOPO085-09, GenBank GU669597, (CCDB, USNM). Sandoval Co: Pakitza Campground, 4 mi. E. Ponderosa, Santa. Fe Nat. Forest: 7 ♂, 9 ♀, em. 4–11 Aug 1969, D. & M. Davis, DRD 642.1,Host: *Robinia neomexicana*, slides USNM 34183, 34184, (USNM).

#### Distribution.

Known only from the southwestern United States from Arizona and New Mexico.

#### Etymology.

The specific name is derived from the specific name of its plant host. The specific epithet is an adjective in the nominative singular.

### Macrosaccus uhlerella

(Fitch) comb. n.

http://species-id.net/wiki/Macrosaccus_uhlerella

Figs 7, 8 28, 29–31 51–53 Tables 1 2

Argyromiges uhlerella Fitch, 1859:838, No. 337.
Lithocolletis uhlerella (Fitch).- Chambers 1871: 183.- Walsingham 1889: 53.- Riley 1891: 109, No. 5900.- Dyar 1902 [1903]: 551, No. 6268.- Braun 1908: 291.- Forbes 1923: 192.- Meyrick 1912a: 7; 1912b: 33.- Braun 1914: 114.- McDunnough 1939: 95, No. 9190.
Phyllonorycter uhlerella (Fitch).- Ely 1918: 59.- Davis 1983: 10.- De Prins and De Prins 2005: 360.- De Prins and De Prins 2011.
Lithocolletis amorphaeella Chambers 1877: 132, 137.- Walsingham 1889: 53 (synonym of *Lithocolletis uhlerella*)*.-*Riley 1891: 109, No. 5900.- Braun 1908: 292.- Dyar 1902 [1903]: 551, No. 6268.- Braun 1908: 291.- Meyrick 1912a: 7; 1912b: 33.- Braun, 1914: 114.- McDunnough 1939: 95, No. 9190.
Lithocolletis amorphae Frey and Boll 1878: 275.- Walsingham 1889: 53 (synonym of *Lithocolletis uhlerella*).*-*Riley 1891: 109, No. 5900.- Dyar 1902 [1903]: 551, No. 6268.- Braun 1908: 292.- McDunnough 1939: 95, No. 9190.

#### Diagnosis.

**Figures 28–31.**
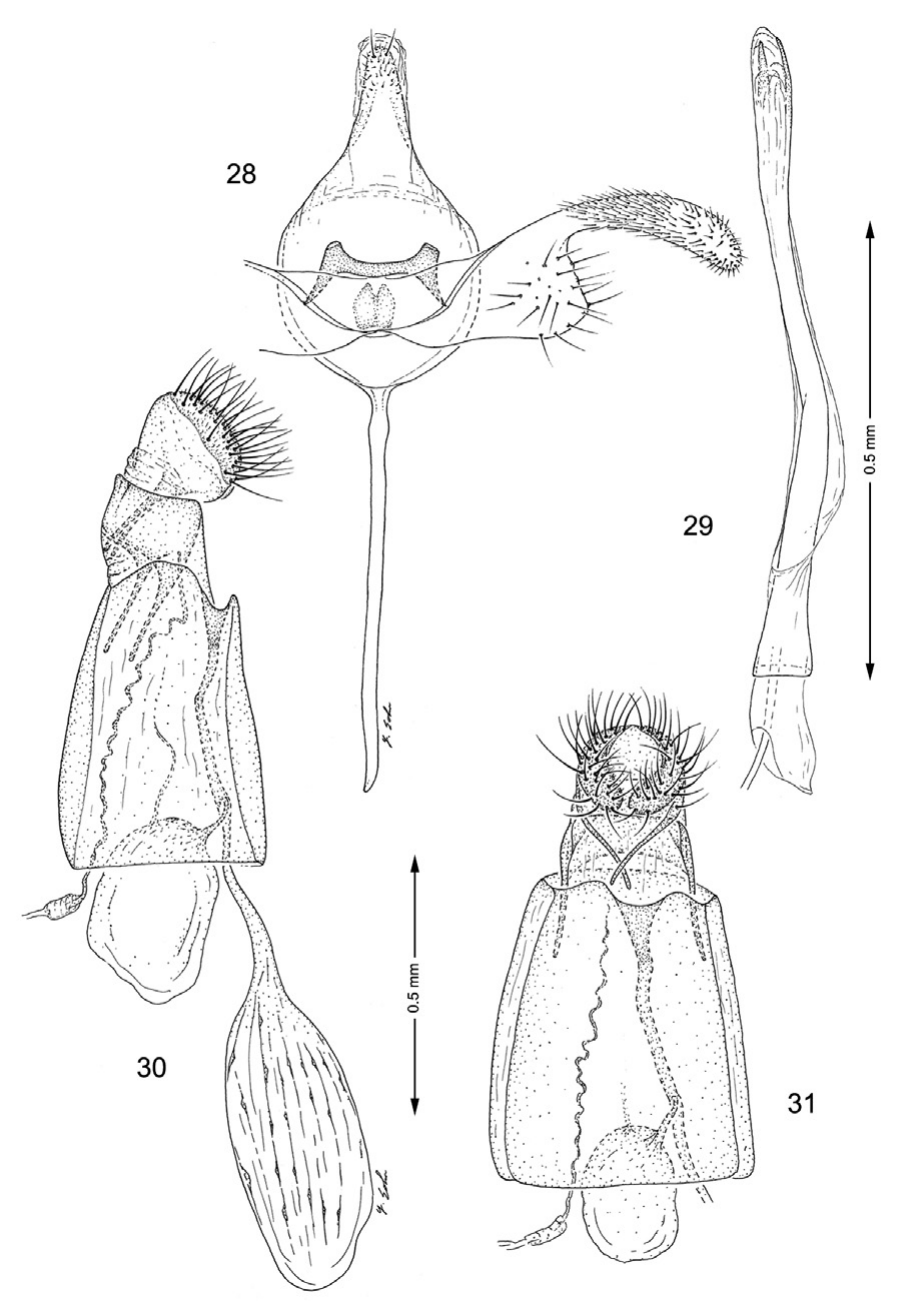
Genitalia**,** *Macrosaccus uhlerella*. **28–29** Male. **28** Genital capsule, ventral view **29** Aedeagus **30–31** Female. **30** Lateral view **31** Segments 7–10, ventral view.

The forewing pattern of this species is most similar to that of *Macrosaccus morrisella* in having the basal strigulae less oblique than those present in *Macrosaccus robiniella* and *Macrosaccus neomexicanus*, but it differs from *Macrosaccus morrisella* in lacking the distinct basal white streak typical of the latter. The male genitalia of *Macrosaccus uhlerella* are distinct in possessing the most modified, slender valvae (Fig. 28) of any member of *Macrosaccus*.

#### Adult

(Figs 7, 8). Forewing length 2.2–2.8 mm.

*Head:* Vestiture of head and antenna similar to *Macrosaccus robiniella* and *Macrosaccus morrisella*.

*Thorax*: Light to dark brown to fuscous dorsally, sometimes with a slight orange luster and a suffusion of fuscous posteriorly; shiny white ventrally; tegula usually orange brown, occasionally with fuscous suffusion posteriorly. Forewing mostly light brownish orange with 4 white costal strigulae, each usually with pale to dark fuscous borders; pattern similar to *Macrosaccus morrisella* except without a distinct slender, oblique, white streak from tegula at base of wing; 3 white dorsal strigulae usually present, but these oriented less obliquely than in *Macrosaccus robiniella*; basal strigula white, but sometimes obscure; median strigula connected to second costal strigula to form a narrow white fascia as in 4; dorsal half of wing with black scaling variably present between strigulae; a large black apical spot present similar to that of *Macrosaccus robiniella* and *Macrosaccus morrisella*; cilia light grey to white. Hindwing, including fringe, uniformly grey. Legs similar to *Macrosaccus robiniella* in colour pattern.

*Abdomen:* Similar to *Macrosaccus robiniella*, dark fuscous dorsally and white ventrally with greyish suffusion laterally on anterior portion of segments 2–7 and sometimes ventrally on A8.

*Male genitalia* (Figs 28, 29): Distal half of valva abruptly constricted to ~ 1/3 the width of basal half; saccular lobe broadly produced, truncate. Saccus a long, slender rod ~ 1.3× length of valva. Aedeagus long and slender, ~ 3.0× length of valva, with phallobase only slightly more enlarged than aedeagus.

*Female genitalia* (Figs 30, 31): Ductus bursae long and slender, slightly longer (~ 1.2×) than length of elongate corpus bursae. Accessory bursae spherical, ~ half the length of corpus bursae, arising from approximately midway along ductus bursae. Corpus bursae elliptical, with series of small, dentate spicules arranged in faint longitudinal folds or striae; walls of anterior end (distal 1/6) of corpus bursae membranous.

#### Larva and pupa.

Not examined.

#### Larval mine

(Figs 51–53). The mature mine is an elongate-oval, whitish blotch located on the under (abaxial) side of the leaf usually near the edge of the leaflet. Eventually, as the mine becomes tentiform, the leaf edge is slightly curled (Fig. 51).

#### Hosts.

(Table 1). *Amorpha fruticosa* L., (Chambers 1878: 58; Frey and Boll 1878: 276; Walsingham 1889: 119; Braun 1908: 292). *Amorpha* sp., *Robinia* sp. (Robinson et al. 2002: 357). The primary host, *Amorpha fruticosa*, is a shrub up to 4 meters high which occurs naturally from Louisiana to Florida and north to Wisconsin and Pennsylvania. Because no adults reared from *Robinia* are known or have been examined, the host record reported in Robinson et al. (2002) is questionable.

#### Types.

*Argyromiges uhlerella* Fitch [type material and deposition not stated, believed lost]: [New York]. *Lithocolletis amorphaeella* Chambers: Lectotype (present designation), ♀: “Type 1327; Chambers, Colorado; *Lithocolletis amorphaeella* Cham.; Lectotype ♀, *Lithocolletis amorphaeella* Chambers, by D. Davis; ♀ genitalia on slide 4530, D.R. Davis”, (MCZ), [head, right wings missing]. *Lithocolletis amorphae* Frey and Boll: Type material not stated, deposition unknown; [Texas].

#### Material examined.

UNITED STATES: COLORADO: Specific locality unknown: 1 ♀, lectotype, *Lithocolletis amorphaeella* Cham., DRD slide 4530, (MCZ). ILLINOIS: McDunnough Co: 3 mi. East of Good Hope, Short Fork Seep, Ti7N-R2W, Section 27–28: 2 ♂, 2 ♀, 7 Aug 2010, emerged 11–17 Aug 2010, leaf mine on *Amorpha fruticosa*, J. Wiker and T. Harrison, slide USNM 33918, (USNM). Putnam Co: 1 ♂, 1 ♀, 29 Mar 1938, DRD slide 4508; 1 ♂, 18 Aug 1939; 1 ♂, 8 Sep 1940, DRD slide 4507; 2 ♂, 20 Sep 1967, reared from underside leafmine *Amorpha fruticosa*; 2 ♂, 25 Sep 1940; 1 ♂, 5 Oct 1946, DRD slide 4506, reared from leafmine, *Amorpha fruticosa*, M. O. Glenn, (INHS). Vermilion Co: Kickapoo State Recreation Area: 1 ♀, 13 Jun 1991, moth iss. 24 Jun 1991, T. Harrison, (INHS). MISSOURI: Boone Co: Colombia: 1 ♀, 14 Dec 1969, W. S. Craig, under bark of sycamore, (USNM). TEXAS: Specific locality unknown: 4 ♀, from *Amorpha fruticosa*, (BMNH). Dallas Co: Dallas: 2 ♂, 3 ♀, Boll, slide BM 4003, (BMNH); Dallas, Boll. 1876, 1 ♂, 4 ♀, bred from *Amorpha fruticosa*, Stainton collection 1893-134, (BMNH).

#### Distribution.

*Macrosaccus uhlerella* is known to occur from Colorado, Illinois, Missouri, New York, and Texas.

#### Remarks.

For over 120 years *Argyromiges uhlerella* Fitch has been regarded as the senior synonym of *amorphaeella* Chambers. The inadequacy of the original description of *Argyromiges uhlerella* (quoted below), together with the disappearance of any type material, has caused some uncertainty regarding this insect’s identity. In his review of the insects feeding on *Robinia pseudoacacia*, Fitch (1859) proposed two names now assigned to *Macrosaccus* which he believed to be related to the *Robinia* leafminer: *Argyromiges pseudacaciella* (now considered a synonymn of *Macrosaccus robiniella*) and *Argyromiges uhlerella*. Fitch stated that he did not know the hosts for these two moths (i.e., neither had not been reared). Walsingham (1889) concluded that Fitch’s description of *Macrosaccus uhlerella* agreed with that of *Macrosaccus amorphaeella*, proposed 18 years later by Chambers (1877), and he consequently synonymized the latter name. All later workers treating this complex accepted Walsingham’s decision. Closer examination of wing pattern variation within large series of reared *Macrosaccus robiniella*, however, suggests that Fitch’s description more approximates the greyish forewing colour of some specimens of *Macrosaccus robiniella* than it does the non-greyish, more brownish forewing colour prevalent in the smaller sample of adult *Macrosaccus* rearedfrom *Amorpha fruticosa* examined. Fitch’s description also contains such abnormalities as the “five white spots along their outer sides” and “the tip of the wings is here replaced by a short black stripe thrice as long as it is wide”, both of which may possibly reflect the poor condition of his specimen(s). Worn specimens of *Macrosaccus robiniella* have been observed with one or two strigulae indistinct or missing, as well as some with reduced apical spots. Similar variation might also be found to occur within moths reared from *Amorpha*, once more specimens become available for examination.

No type material of *“Argyromiges” uhlerella* is believed to exist. In 1977, during a search for Fitch’s Lepidoptera types deposited in the USNM, Tim McCabe found a pin bearing Fitch’s label 8158 (the type number for *uhlerella*) in the main collection. The moth was missing and was presumed destroyed. The pin with that number has since disappeared. Because the name *uhlerella* has been used consistently as the valid name for this taxon since before 1899, we believe that this usage should continue even though some doubt now exists regarding the correct application of the name.

#### Original description of 

Argyromiges uhlerella

####  Fitch:

**“337.** Uhler’s leaf-miner, *Argyromiges* *Uhlerella*, new species.”

“This resembles *Pseudacaciella*, but is throughout of paler color, the fore wings being golden gray, with five white spots along their outer sides, of which the hindmost ones are small, the others quite large and bordered with blackish upon their anterior sides; and the black dot on the tip of the wings is here replaced by a short black stripe thrice as long as it is wide; whilst the hind wings and their fringes are pale silvery gray. These marks will suffice to distinguish this from the two preceding species.”

### Macrosaccus gliricidius

Davis sp. n.

urn:lsid:zoobank.org:act:19A1403C-D313-42BE-AE90-588AF6876F0D

http://species-id.net/wiki/Macrosaccus_gliricidius

Figs 1 9 32–35 54–58 Tables 1 2

#### Diagnosis.

The forewing pattern of this species is similar to *Macrosaccus robiniella* and *Macrosaccus neomexicanus* in possessing 4 white costal and 3 dorsal, mostly sharply oblique strigulae, with a median fascia often formed by the junction of the 2nd costal and median dorsal strigulae. The pale golden brown ground colour of *Macrosaccus gliricidius* is distinctly paler than that of the other species. The forewing of *Macrosaccus gliricidius* also differs from other *Macrosaccus* in possessing a small, elongate white subapical spot and a more reduced dark fuscous apical spot. The male valva of *Macrosaccus gliricidius* is distinct in having the distal half more broadly rounded than that of other *Macrosaccus*.

#### Adult

(Fig. 9). Forewing length 2.2–2.6 mm.

*Head:* Vestiture of head and antenna similar to *Macrosaccus robiniella* except vertex generally paler and with more white scales concentrated toward occiput.

*Thorax:* Dorsum with a narrow, median, longitudinal band of light golden brown bounded laterally with white; tegula light golden brown; venter white. Forewing pale golden brown with 4 equally spaced, oblique, white costal strigulae and 3 white, dorsal strigulae, each bordered by dark brown scales; 2nd costal strigula connected to median dorsal strigula; subapical dorsal strigula directed inward toward small, white subapical spot; dark fuscous apical spot poorly developed, with a more elongate subapical spot immediately basad to rudimentary apical spot; fringe mostly pale greyish white, with narrow, dark brown median band and broad, grey inner band. Hindwing, including fringe, uniformly grey. Foreleg mostly dark fuscous dorsally, white ventrally, with 2 white annuli around basal tarsomeres; midleg mostly white with oblique bands of white extending dorsally over tibia; tarsomeres more broadly banded with white dorsally; hindleg mostly white with much of tibia fuscous dorsally, and with 3 broad fuscous annuli dorsally over tarsomeres.

*Abdomen:* dark brown dorsally and laterally along anterior margins of A3–7; white ventrally.

**Figures 32–35.**
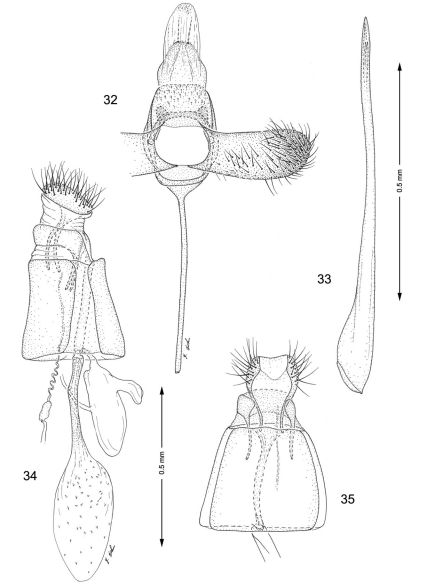
Genitalia**,** *Macrosaccus gliricidius*. **32–33** Male. **32** Genital capsule, ventral view **33** Aedeagus **34–35** Female. **34** Lateral view **35** Segments 7–10, ventral view.

*Male genitalia* (Figs 32, 33): Valva simple, becoming slightly broader near apex; apex broadly rounded, setose; saccus a slender, elongate rod ~ 1.6× length of valva. Aedeagus very long and slender, ~ 3.5× length of valva; phallobase moderately enlarged.

**Figures 36–40.**
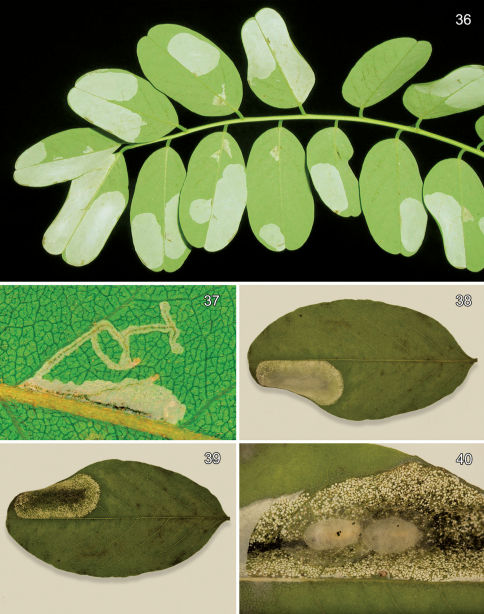
Leafmines of *Macrosaccus robiniella* on *Robinia pseudoacacia*. **36** Abaxial blotch mines, with kind permission of György Csóka **37** Early instar, abaxial serpentine mines, with kind permission of György Csóka **38** Abaxial blotch mine **39** Adaxial view of Fig. 38 **40** Opened mine with 2 cocoons.

**Figures 41–44.**
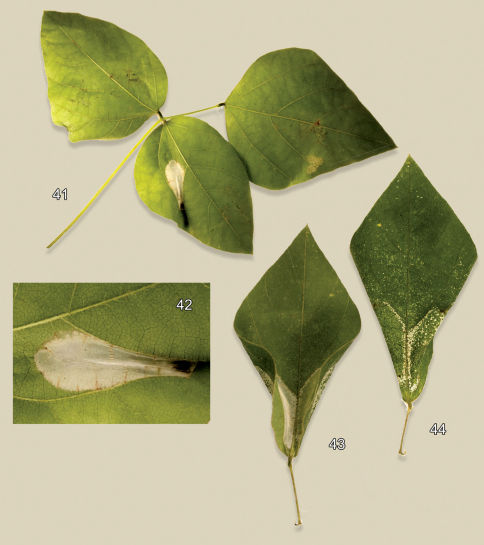
Leafmines of *Macrosaccus morrisella* on *Amphicarpa bracteata*. **41** Abaxial tentiform blotch mine **42** Detail of abaxial tentiform blotch mine **43** Two abaxial blotch mines at leaf base **44** Adaxial view of Fig. 43.

**Figures 45–50.**
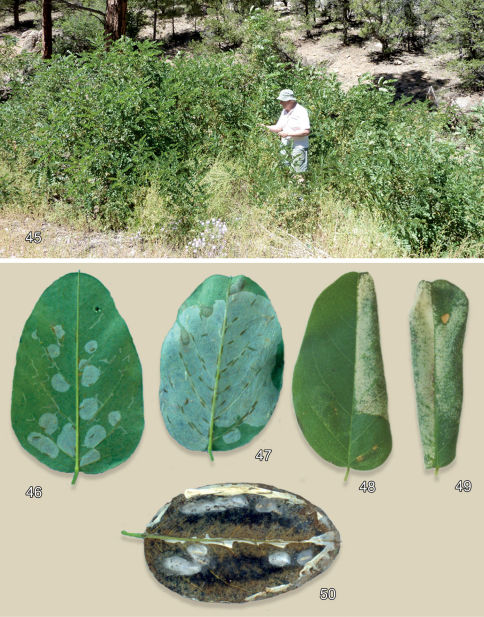
Habitat andleafmines of *Macrosaccus neomexicanus* on *Robinia neomexicana*. **45** Mixed pine-juniper habitat of *Robinia neomexicana*, Kaibab National Forest, Coconino Co., Arizona, ~2130 m **46** Multiple early instar serpentine and blotch mines on abaxial leaf surface **47** Later stage abaxial mines after multiple blotch mines begin to coalesce **48** Late stage tentiform blotch mine, adaxial view **49** Completely folded leaf resulting from double tentiform mines, adaxial view **50** Opened (with ventral leaf epidermis removed) aggregate blotch mines with 8 pupal cocoons, abaxial view.

**Figures 51–53.**
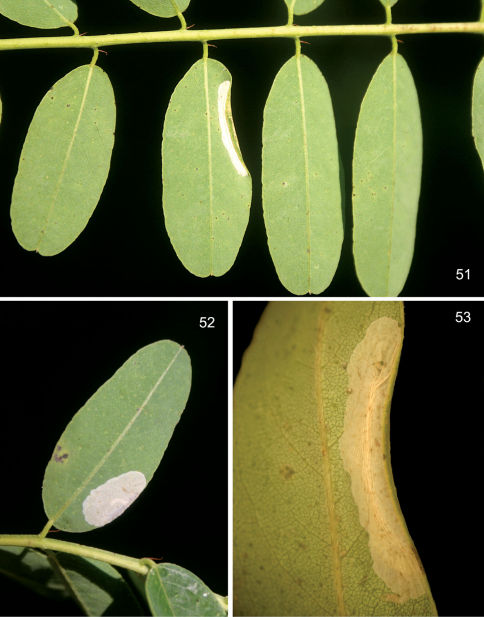
Leafmines of *Macrosaccus uhlerella* on *Amorpha fruticosa*. **51** Abaxial tentiform blotch mine **52** Flat blotch mine **53** Detail of abaxial tentiform blotch mine. Photographs by T. Harrison.

*Female genitalia* (Figs 34, 35): Ductus bursae long and slender, ~ 1.25× the length of corpus bursae. Accessory bursae ~ 0.8× the length of corpus bursae, arising from near middle of ductus bursae at a point where the ductus is slightly constricted; a smaller lateral pouch arising near caudal end of accessory bursae. Corpus bursae elliptical, with numerous acute spicules somewhat evenly scattered over much of inner surface but less dense near anterior end.

#### Larva and pupa.

(Figs 56, 58).Similar to that of *Macrosaccus robiniella*.

#### Larval mine

**Figures 54–58.**
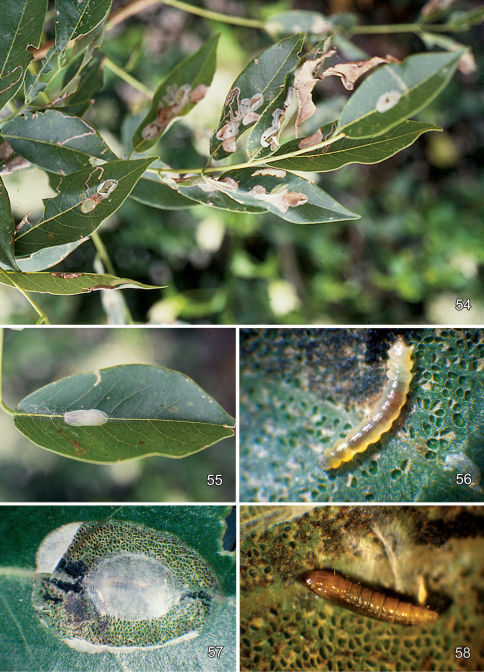
Leafmines of *Macrosaccus gliricidius* on *Gliricidia sepium*. **54** General damage to host **55** Adaxial blotch mine **56** Late instar tissue feeding larva **57** Open blotch mine with single cocoon **58** Pupa with cocoon removed. Photographs by R. Cave.

(Figs 54–58).The mine begins as an elongate serpentine track which abruptly enlarges to an elongate-oval, whitish blotch located on either the upper (adaxial) or lower (abaxial) side of the leaflet. When present on the under side, the blotch mines usually develop along the midrib. Only the upper side blotch mines occurred directly on top of the midrib (Fig. 55, Cave, *in litt*.).

#### Host.

(Table 1). Fabaceae: *Gliricidia sepium* (Jacq.). *Gliricidia sepium* isa small to medium-sized, thornless tree growing to a height of 10–12 meters. It is believed to have originated in Central America and has been introduced into many tropical countries around the world. It can be grown as dense hedges and is frequently used as “living fences”.

#### Parasitoids.

Eulophidae: *Zagrammosoma multilineatum* (Hansson & Cave, 1993).

#### Holotype.

**♂:** HONDURAS: Dept. Francisco Morazán: Guaimaca, Rio Morazán, 14°32'N, 86°51'W: 26 Jul 1992, em. 5 Aug 1992, R. D. Cave, DRD1165, Host: *Gliricidia sepium*, digital image captured, (USNM).

#### Paratypes.

HONDURAS: Dept. Francisco Morazán: Guaimaca, Rio Morazán, 14°32'N, 86°51'W: 2 ♂, 4 ♀, 26 Jul 1992, em. 5 Aug 1992, R. D. Cave, DRD1165, Host: *Gliricidia sepium*, slides USNM 34118–34121, BOLD ID RDOPO086-09, GenBank GU669594, BOLD ID RDOPO087-09, GenBank GU669595, (USNM). San Antonio de Oriente, El Zamorano: 1 ♀, 21 Jul 1988, R. Cave, Host: *Gliricidia sepium*, (USNM). Tegucigalpa: Steven Passoa, (USNM). Dept. Olancho: Juticalpa, Sta. Cruz: 2 ♂, 1 ♀, 5 Aug 1988, R. Cave, Host: *Gliricidia sepium*, slides USNM 30888, 30889, 30893, (USNM). FRENCH WEST INDIES: GUADELOUPE: Lamentin, Chaude Ravine: 1 ♂, 10 ♀, 10 May 2004, Jean Etienne, Host: *Gliricidia sepium*, slides USNM 34186, 34187, 34265, DNA/BOLD ID RDOPO366-10, GenBank HM382079, DNA/BOLD ID HM382080, GenBank HM382080, (USNM).

#### Distribution.

Known from Central America (Honduras) and the West Indies (Guadeloupe).

#### Etymology.

The species name is derived from the generic name of its host, *Gliricidia*. The specific epithet is a noun in the nominative singular.

**Figures 59–65.**
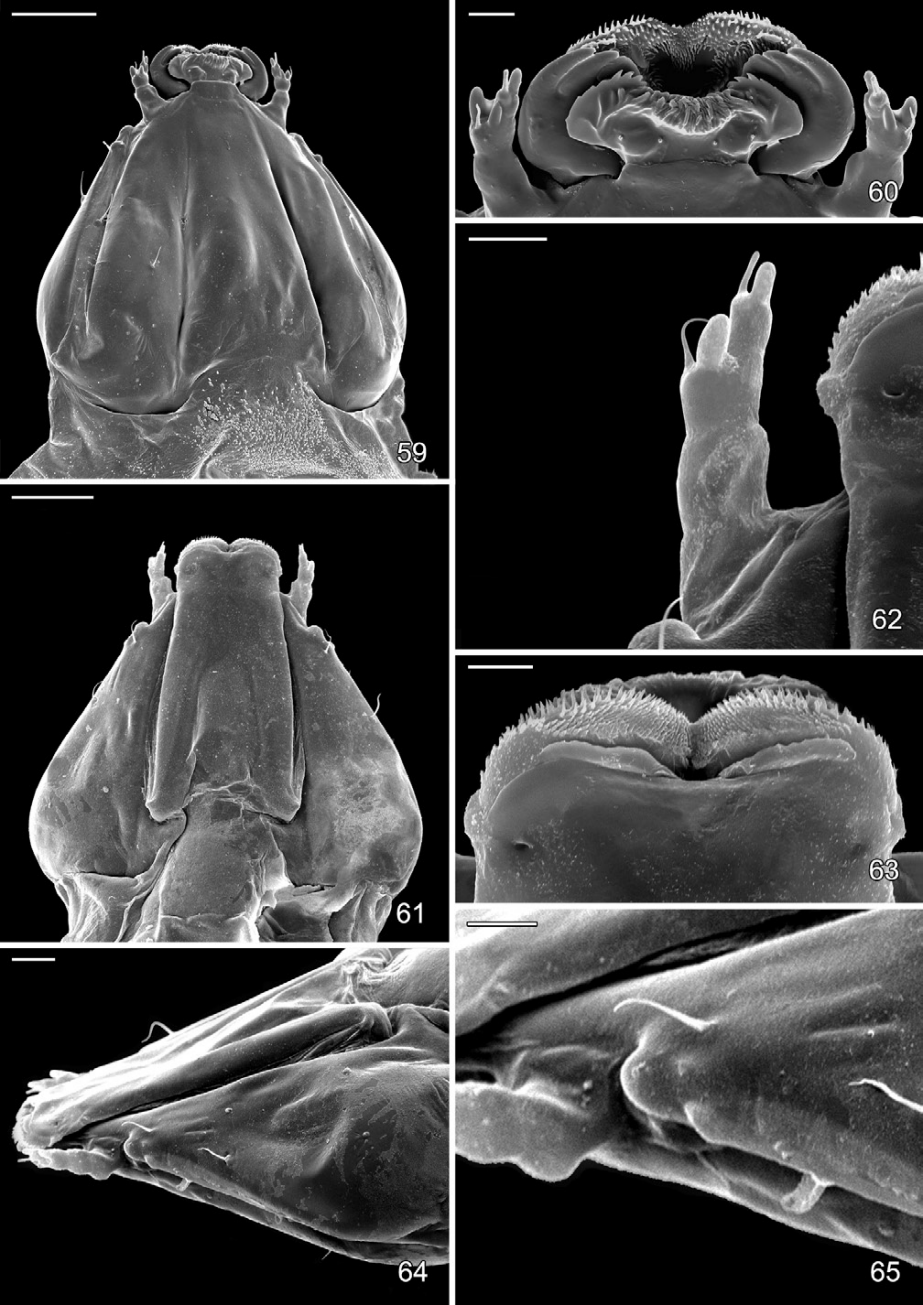
Sap feeding larval instar of *Macrosaccus robiniella*. **59** Head, dorsal view (50 µm) **60** Detail of mouthparts, antenna, dorsal view (10 µm) **61** Head, ventral view (50 µm) **62** Antenna dorsal view (10 µm) **63** Detail of mouthparts, ventral view (10 µm) **64** Head, lateral view (20 µm) **65** Detail of stemmatal area, lateral view (20 µm). (Scale lengths in parentheses).

**Figures 66–71.**
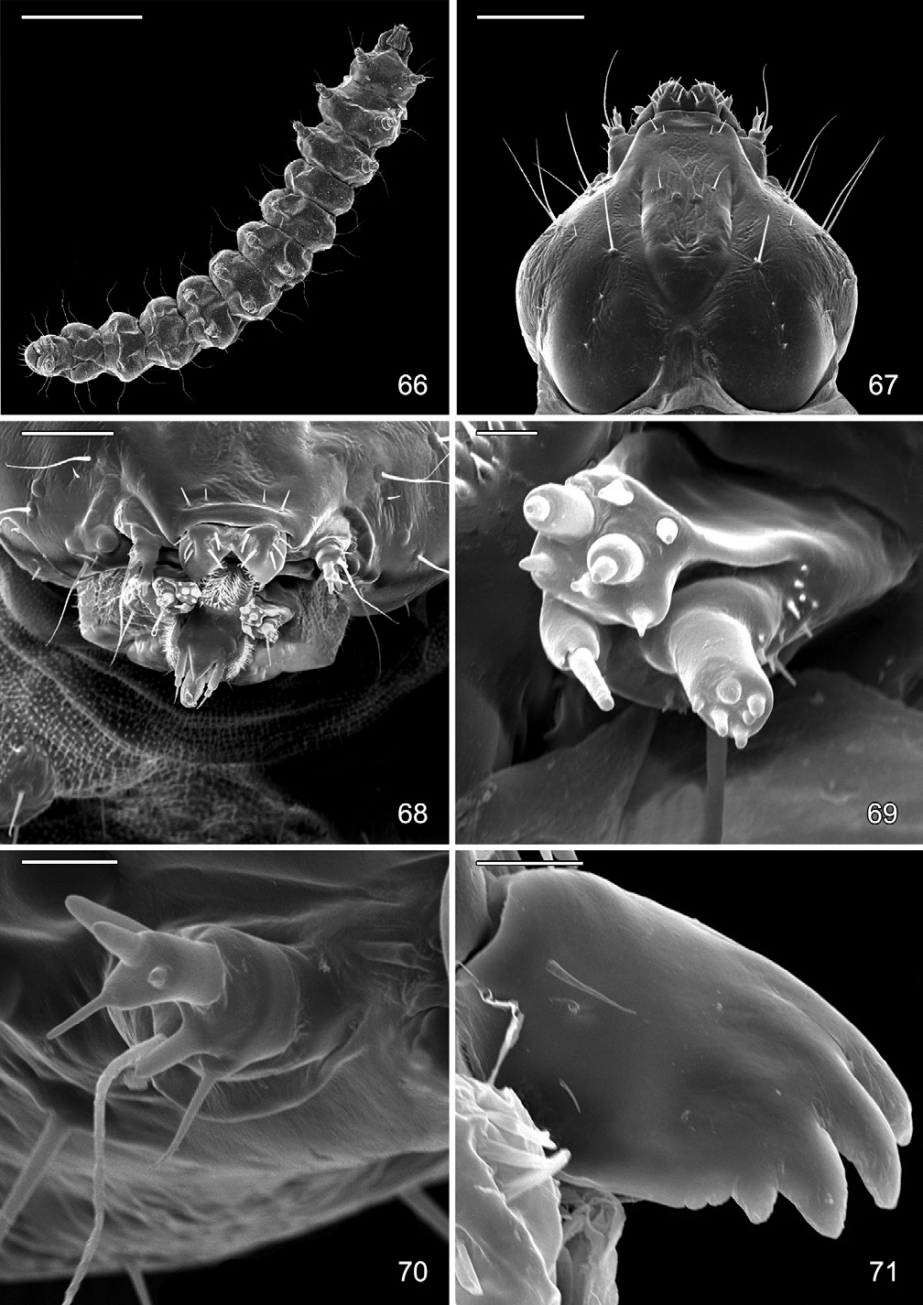
Late tissue feeding instar larva of *Macrosaccus robiniella*. **66** Ventral view (1 mm) **67** Head, dorsal view (100 µm) **68** Mouthparts, dorsal view (50 µm) **69** Maxilla, anterior view (5 µm) **70** Antenna, lateral view (10 µm) **71** Mandible, dorsal view (10 µm). (Scale lengths in parentheses).

**Figures 72–77.**
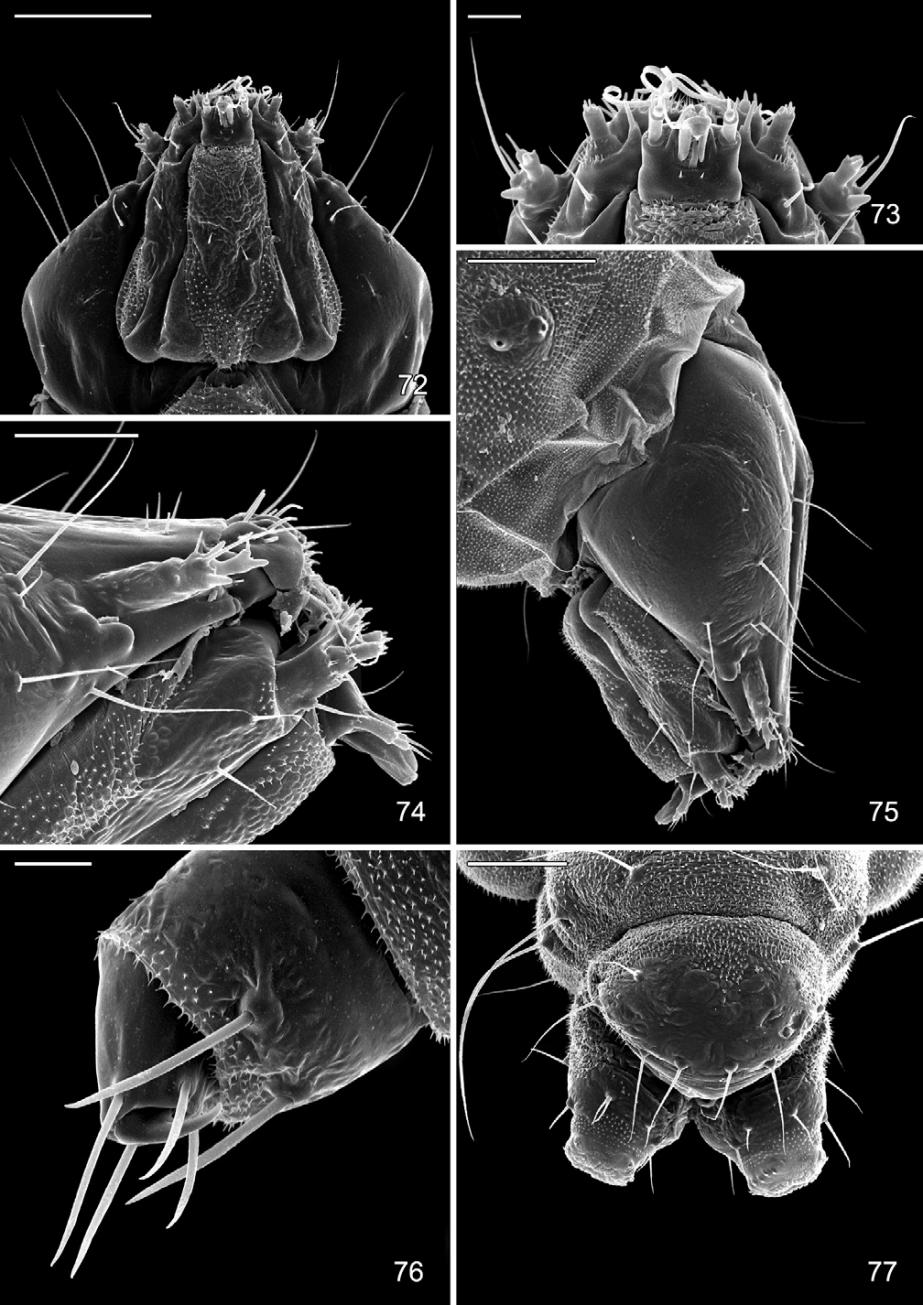
Late tissue feeding instar larva of *Macrosaccus robiniella*. **72** Head, ventral view (100 µm) **73** Detail of mouthparts, ventral view (20 µm) **74** Lateral view of mouthparts (50 µm) **75** Lateral view of head (100 µm) **76** Thoracic leg (20 µm) **77** Abdominal segments 9, 10, dorsal view (100 µm). (Scale lengths in parentheses).

**Figures 78–83.**
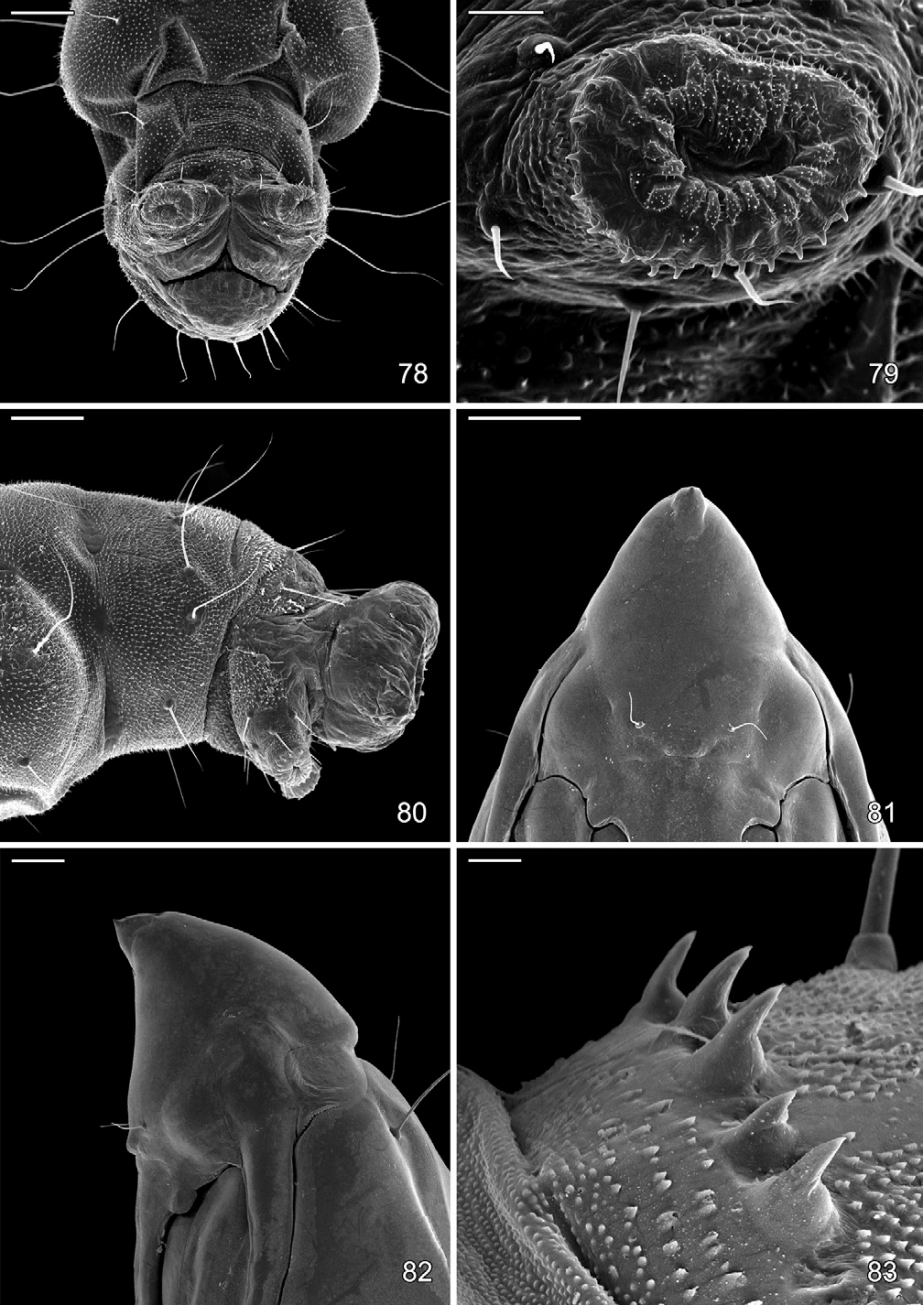
Late tissue feeding instar larva and pupa of *Macrosaccus robiniella*. **78–80** Larva **78** Abdominal segments 9, 10, ventral view **79** Anal proleg **80** Abdominal segments 9, 10, lateral view. **81–83** Pupa **81** Head, ventral view **82** Lateral view **83** Anterior row of dorsal abdominal spines. (Scale lengths in parentheses).

**Figures 84–89.**
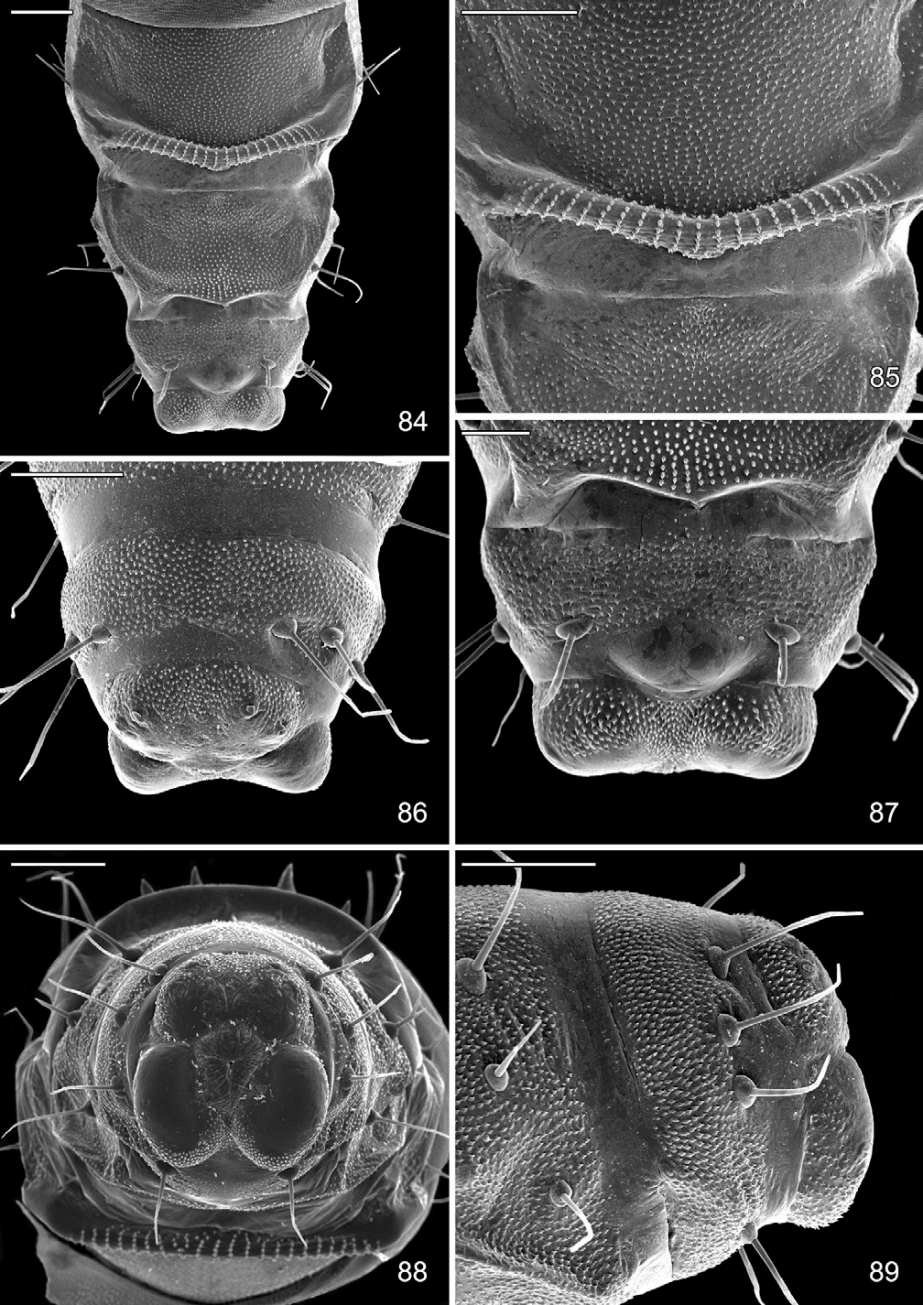
Pupa of *Macrosaccus robiniella*. **83** Abdominal segments 7, 8, 9+10, ventral view (100 µm) **85** Detail of accessory cremaster, abdominal sternum 7 (100 µm) **86** Abdominal segments 8, 9+10, dorsal view (100 µm) **87** Abdominal segments 8, 9+10, ventral view (50 µm) **88** Caudal view of abdomen (100 µm) **89** Abdominal segments 8, 9+10, lateral view (100 µm). (Scale lengths in parentheses).

**Figures 90–98.**
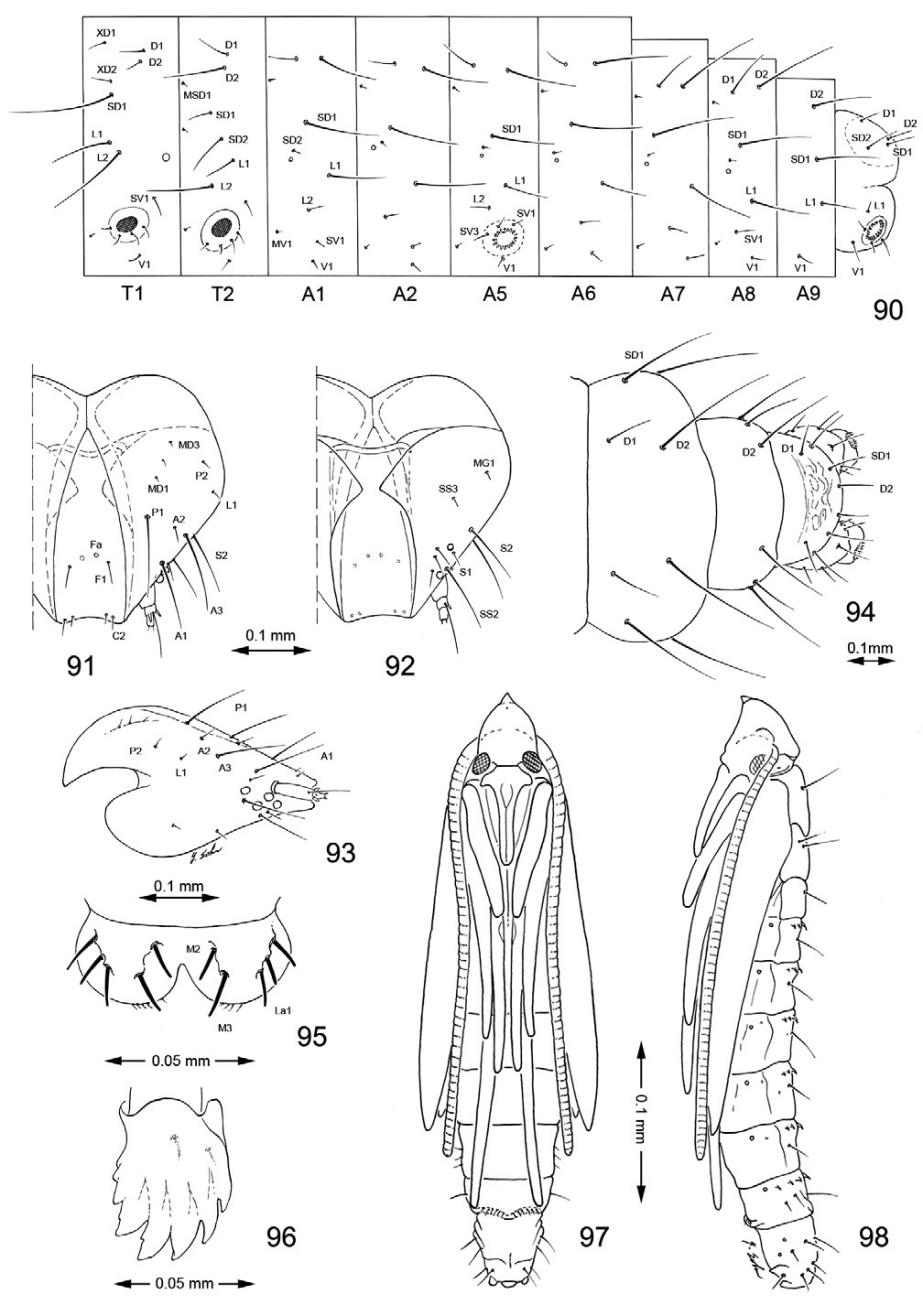
Late tissue feeding instar larva and pupa of *Macrosaccus robiniella*. **90–96** Larval chaetotaxy. **90** Lateral schematic of prothorax, mesothorax, and abdominal segments 1, 2, 5–10 **91** dorsal view of head **92** Ventral view **93** Lateral view **94** Dorsal view of abdominal segments 8–10 **95** Labrum, dorsal view **96** Mandible. **97–98** Pupa. **97** Ventral view **98** lateral view. (Bar scale for figures as indicated).

## Supplementary Material

XML Treatment for Macrosaccus

XML Treatment for Macrosaccus robiniella

XML Treatment for Macrosaccus morrisella

XML Treatment for Macrosaccus neomexicanus

XML Treatment for Macrosaccus uhlerella

XML Treatment for Macrosaccus gliricidius
